# Ultra-thin fluorocarbon foils optimise multiscale imaging of three-dimensional native and optically cleared specimens

**DOI:** 10.1038/s41598-019-53380-2

**Published:** 2019-11-21

**Authors:** Katharina Hötte, Michael Koch, Lotta Hof, Marcel Tuppi, Till Moreth, Monique M. A. Verstegen, Luc J. W. van der Laan, Ernst H. K. Stelzer, Francesco Pampaloni

**Affiliations:** 1 Physical Biology Group, Buchmann Institute for Molecular Life Sciences (BMLS), Goethe-Universität Frankfurt am Main, D-60438 Frankfurt am Main, Germany; 2 Institute of Biophysical Chemistry and Center for Biomolecular Magnetic Resonance and Cluster of Excellence Macromolecular Complexes (CEF), Goethe University, Frankfurt/Main, Germany; 3 Department of Surgery, Erasmus MC – University Medical Center, Rotterdam, The Netherlands; 4 Present Address: The Francis Crick Institute, London, NW11ST, UK

**Keywords:** Light-sheet microscopy, Multicellular systems

## Abstract

In three-dimensional light microscopy, the heterogeneity of the optical density in a specimen ultimately limits the achievable penetration depth and hence the three-dimensional resolution. The most direct approach to reduce aberrations, improve the contrast and achieve an optimal resolution is to minimise the impact of changes of the refractive index along an optical path. Many implementations of light sheet fluorescence microscopy operate with a large chamber filled with an aqueous immersion medium and a further inner container with the specimen embedded in a possibly entirely different non-aqueous medium. In order to minimise the impact of the latter on the optical quality of the images, we use multi-facetted cuvettes fabricated from vacuum-formed ultra-thin fluorocarbon (FEP) foils. The ultra-thin FEP-foil cuvettes have a wall thickness of about 10–12 µm. They are impermeable to liquids, but not to gases, inert, durable, mechanically stable and flexible. Importantly, the usually fragile specimen can remain in the same cuvette from seeding to fixation, clearing and observation, without the need to remove or remount it during any of these steps. We confirm the improved imaging performance of ultra-thin FEP-foil cuvettes with excellent quality images of whole organs such us mouse oocytes, of thick tissue sections from mouse brain and kidney as well as of dense pancreas and liver organoid clusters. Our ultra-thin FEP-foil cuvettes outperform many other sample-mounting techniques in terms of a full separation of the specimen from the immersion medium, compatibility with aqueous and organic clearing media, quick specimen mounting without hydrogel embedding and their applicability for multiple-view imaging and automated image segmentation. Additionally, we show that ultra-thin FEP foil cuvettes are suitable for seeding and growing organoids over a time period of at least ten days. The new cuvettes allow the fixation and staining of specimens inside the holder, preserving the delicate morphology of e.g. fragile, mono-layered three-dimensional organoids.

## Introduction

Light Sheet Fluorescence Microscopy (LSFM) has revolutionized the imaging of three-dimensional specimens ranging from micrometres (spheroids, organoids) to centimetres (biopsies, model organisms) in diameter^1–4^. Light spots and light sheets based on conventional light sources have been used for more than one century. An excellent example is Zsigmondy’s ultramicroscope for the study of the physicochemical properties of colloids^5^. However, lasers are required for the true optical sectioning capability. While laser-based light sheet–based macroscopes had been built several times^6^, the performance of light sheet at a microscopic level was not known until Huisken *et al*.,^7^ described a diffraction-limited laser light sheet–based fluorescence microscope, applied it to live biological samples and evaluated its applicability for multiple-view imaging^8,9^. In 2007, SPIM was applied for the imaging of 3D cell cultures in collagen gel and of multicellular tumor spheroids^8^. The same year, Dodt *et al*. used a light sheet microscope to image large optically cleared specimens, including mouse brain^10^. The introduction of the Digitally-scanned Light Sheet Microscope (DSLM) replaced the static light sheet generated with a cylindrical lens with a scanned light sheet, resulting in a more flexible set-up with increased penetration depth and less illumination artefacts^11^. A further milestone, the invention of lattice light sheet microscope, opened LSFM to super-resolution capabilities^12^. Currently, a large diversity of LSFM setups exist for application in cell and developmental biology, pathology, neuroscience, and biophysics^13–15^.

Ever since the introduction of LSFM, agarose and other hydrogels have been used as transparent and biocompatible mounting media to immobilise specimens during imaging^2,16,17^. While agarose-gel embedding is convenient for mounting drosophila and zebrafish embryos^7,11^, it is not suitable for most 3D cell cultures and generally inapplicable for mounting optically cleared specimens. In 3D-cultures such as organoids, cells grow in soft hydrogels, e.g. Matrigel^18^, which are composed of extracellular matrix (ECM) proteins such as collagen and laminin^19^. Embedding 3D-cultures into agarose gels deforms both the ECM hydrogels as well as the delicate multi-cellular structures growing inside. Attempts have been made to avoid specimen embedding, e.g. by depositing specimens into pre-cast “agarose beakers”^20,21^. However, the “agarose-beaker” mounting technique still presents many drawbacks including a time-consuming, laborious fabrication procedure and mechanical instability. Most importantly, soluble components rapidly diffuse in and out of the agarose gel, implying that the specimens cannot be properly isolated from potential contaminants of chemical or bacterial origin floating in the LSFM sample chamber^20^.

In order to reduce strong light scattering effects caused by refractive index mismatches in three-dimensional dense tissues, optical clearing solutions are used to homogenise the refractive indices across the whole sample. The impulse given by LSFM led to the ongoing development of new clearing protocols in the last decade. Dodt *et al*. combined the century-old Spalteholz/BABB optical clearing method^22,23^ with LSFM, obtaining 3D reconstructions of the mouse brain^10^. Currently, two major families of optical clearing solutions are used: (1) organic solvents-based and (2) water-based clearing solutions^24,25^. Solvent-based optical clearing methods are e.g. BABB (benzyl alcohol and benzyl benzoate), uDISCO^26^ and Ethanol-ECi^27^. Many water-based clearing protocols have been developed since 2011. These methods can be distinguished in (1) hydrogel embedding-based, such as CLARITY^28^, (2) immersion-based (for which the refractive index matching is achieved just by immersion), e.g. SeeDB^29^, and (3) hyperhydration-based, such as e.g. CUBIC1, and CUBIC2^30^. All these clearing protocols have drawbacks, and have been optimized for specific organs. Comparative studies assessed the applicability range of several methods^31,32^.

Organic solvents for optical clearing are often chemically aggressive and damage the objective lenses or the LSFM sample chamber. Thus, a common approach uses large glass or quartz cuvettes to contain the clearing medium, in which the specimen is immersed^33^. For this imaging approach, low magnification air objective lenses or macro lenses with low numerical apertures (NAs) are frequently used^33,34^, which tend to yield relatively low-resolution images. Water-based clearing solutions are less corrosive compared to organic solvents and can be combined with immersion objective lenses with higher NAs, which allow for a higher resolution and a better image quality. Nevertheless, the handling of large volumes of optical clearing solutions can be challenging, e.g. due to high viscosity or crystallisation of some components in the solution.

In a previous publication, we used thin square cross section glass capillaries with an inner side length of about 1 mm for mounting small organically cleared specimens. This allowed us to use immersion objective lenses in combination with less or non-corrosive media to fill the LSFM chamber^35^. Although we successfully applied this method to the *in toto* study of drug-treated spheroids^36^, connecting and sealing the capillary to the holder proved to be time-consuming.

We introduced fluorocarbon foil (fluorinated ethylene propylene, FEP) for LSFM in 2010, when we used FEP-cylinders to investigate the dynamics of microtubule asters in *Xenopus laevis* egg extracts. The FEP-cylinders allowed the observation of 3D microtubule dynamics unhindered by rigid and flat surfaces^37^. In developmental biology, FEP-cylinders with an 800 µm inner diameter and a 400 µm wall thickness filled with low concentrations of agarose (0.1%) or methylcellulose (3%) provided a more physiological confinement for the long-term live imaging of zebrafish development^38^.

Here, we describe how to fabricate and use ultra-thin FEP-foil cuvettes that eliminate the need for agarose/hydrogel embedding in LSFM imaging. The cuvettes are closed at the bottom end, which greatly simplifies handling compared to the previously used glass capillaries and FEP-cylinders. They allow fast and straightforward specimen mounting using a forceps or pipettes to deposit the specimen into the cuvettes, and facilitate full containment of the internal mounting medium. The ultra-thin FEP-foil cuvettes are compatible with aqueous media as well as with water-based and organic clearing solutions, which all differ in their refractive indices. Compared to agarose embedding, a key advantage of ultra-thin FEP-foil cuvettes is that the clearing medium of choice is confined within the cuvette, whereas the LSFM chamber itself can be filled with harmless and easily handled index-matching media, such as 2,2′-Thiodiethanol (TDE), CUBIC2 or Iodixanol^39–41^. A major advantage of the ultra-thin FEP-foil cuvettes is that they minimise the effect of refractive index mismatch between FEP (n = 1.34) and the surrounding mounting medium (water n = 1.33; TDE n = 1.41; CUBIC2 n = 1.48; Iodixanol n = 1.75) by their reduced wall thickness of only a few microns (~10–12 µm). Our data show that ultra-thin FEP-foil cuvettes allow high quality imaging of native (non-cleared) and optically cleared 3D specimens, such as liver and pancreas organoids, thick murine kidney and brain tissue sections as well as whole murine ovaries. In order to demonstrate the suitability of the cuvette to handle both solvent-based and water-based clearing solutions, we used Ethanol-ECi and CUBIC2 solution, respectively. Their usage is simple of use and they effectively clear our specimens. The image quality allows for the application of multiple-views image reconstruction and for the subsequent application of automated quantitative image analysis pipelines^42,43^.

## Material and Methods

### FEP-foil

FEP-foil was purchased from Lohmann Technologies Ltd, UK (50 µm thickness, Batch No. GRN069662). Fluoroethylene propylene (FEP) is a thermoplastic fluorocarbon polymer composed of the monomers tetrafluoroethylene (CF_2_ = CF_2_) and hexafluoropropylene (CF_2_ = CF-CF_3_). FEP-films have outstanding properties for light microscopy. FEP-films are transparent with a refractive index of 1.341–1.347, which is close to the refractive index of water (1.333; 589.3 nm and 20 °C). Like most fluorocarbon plastics, FEP is chemically inert and resistant to organic solvents, acids and bases. Moreover, the material is autoclavable and biocompatible according to FDA (21CFR.177.1550) and EU (2002/72/EC) requirements (source: DuPont FEP-Fluorocarbon Film – Properties Bulletin).

### Fabrication of positive moulds for vacuum forming

**Figure 1:**
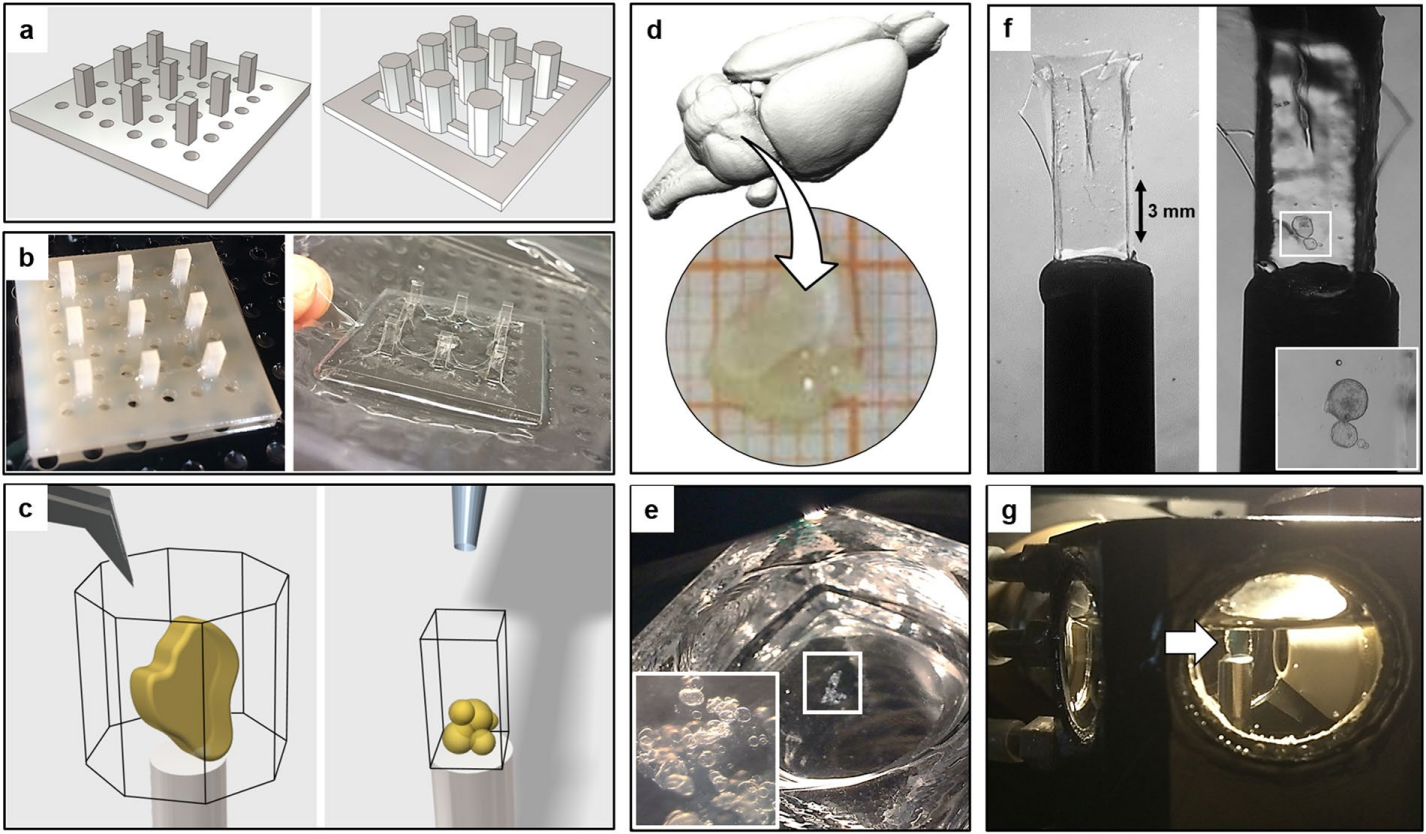
Fabrication of ultra-thin FEP-foil cuvettes and specimen preparation for LSFM. (**a**) CAD-derived drawings of positive moulds for arrays of 3 × 3 cuvettes. (**b**) Printed moulds (left) are used in the vacuum forming process of the ultra-thin FEP-foil cuvettes (right). (**c**) Sample preparation: an organ or a large tissue section (left) is positioned inside an ultra-thin FEP-foil cuvette with a forceps. Smaller samples are deposited by pipetting (right). (**d**) A murine hippocampus section was isolated and optically cleared with CUBIC2. (**e**) Human hepatic organoids, partially embedded in Matrigel, are deposited in an embryo dish before pipetting it into the cuvette. (**f**) Ultra-thin FEP-foil cuvettes before (left) and after (right) human liver organoids are mounted. (**g**) Prior to image acquisition, ultra-thin FEP-foil cuvettes are glued to metal pins for insertion into the mDSLM chamber.

We designed positive moulds of the cuvettes by using the free CAD software “123D Design” (Autodesk Inc., version 2.2.14) (Fig. 1, Supplementary Fig. 1a,b). The 3D drawings were exported to the stereolithographic file format (.stl). The positive moulds were produced by Shapeways (Eindhoven, NL, www.shapeways.com), which uses UV cured acrylic polymer (commercially designed as “ultra-frosted detail”) in a Multijet Modelling (MJM) printing process. Two positive moulds, with arrays of square and octagonal cross section pillars, respectively, were 3D-printed (Fig. 1b). The acrylic polymer has the required mechanical and thermal properties to withstand the vacuum forming process. The MJM printing process allows a detail resolution of 16 µm layer thickness, which is adequate for these moulds. Prior to their usage, the positive moulds are cleaned by immersion in an ultrasonic bath and inspected with a stereomicroscope.

### Cuvette fabrication with vacuum forming

In order to prepare a 3 × 3 array of vacuum-formed ultra-thin FEP-foil cuvettes, a 12 cm × 12 cm square patch of FEP-foil (Type A, general purpose, thickness 50 µm, DuPont de Nemours Int’l SA, Geneva, Switzerland) is clamped into the frame of a vacuum-forming machine (JT-18, Jin Tai Machining Company, Yuyao, PR of China). Once the heater raises the temperature close to the glass transition temperature of the FEP-foil (260–280 °C), the positive mould is promptly placed onto the vacuum-forming machine, the vacuum suction is switched on and the foil is quickly pressed onto the mould (Fig. 1b, Supplementary Fig. 2). Subsequently, the extruded FEP-cuvette array is carefully removed from the mould with forceps, and cleaned with a detergent solution (1% Hellmanex-II in ultrapure water) in an ultrasonic bath for 10 minutes. The individual cuvettes are cut from the array with a scalpel. Finally, individual cuvettes are glued onto stainless steel pins (diameter 3 mm, length 20 mm) using instant glue (Pattex, Repair Extrem) (Fig. 1c,f).

### Specimen preparation

#### Fixed specimens

Murine brain: Adult murine brains from a transgenic strain bred from the C57BL6/J genetic background, and expressing GFP under the control of the Thy-1 promoter (see Feng *et al*.^44^) were kindly provided by Dr. Walter Volknandt, Goethe-Universität Frankfurt, Faculty of Neurobiology and Biosciences. Mice were kept under 12 hours light and dark cycle with food and water ad libitum. Mouse perfusion was performed according to Stefani *et al*.^45^. Briefly, the mice were euthanized by an intraperitoneal injection of ketamine (180 mg/kg of body weight; Ketavet) and xylazine (10 mg/kg of body weight; Rompun) and intracardially perfused with 10 ml of ice-cold physiological saline (0.9% NaCl) followed by perfusion with 150 ml ice-cold 4% paraformaldehyde in phosphate-buffered saline (PBS: 137 mM NaCl, 2.7 mM KCl, 10.1 mM Na_2_HPO_4_, 1.8 mM KH_2_PO_4_, pH 7.4). Removed brains were postfixed over night at 4 °C in the same fixative and cryoprotected with 30% sucrose/PBS for 24–48 h at 4 °C.

Prior to clearing, the brains were washed three times with PBS for 15 minutes, and the hemispheres were separated. Next, the hemispheres were cut into smaller blocks, with a size of approximately 5 mm × 5 mm × 1.5 mm (Fig. 1d). The blocks were incubated with CUBIC2 clearing solution in a reaction tube overnight. Before imaging, the CUBIC2 solution was refreshed, and, using forceps, the blocks were inserted into octagonal ultra-thin FEP-foil cuvettes with a diameter of 5 mm (Fig. 1c).

Murine kidney: The kidney was fixed in 4% PFA in PBS at 4 °C overnight. Prior to clearing, the kidney was washed three times with PBS for 15 minutes and cut in small blocks.

**Figure 2:**
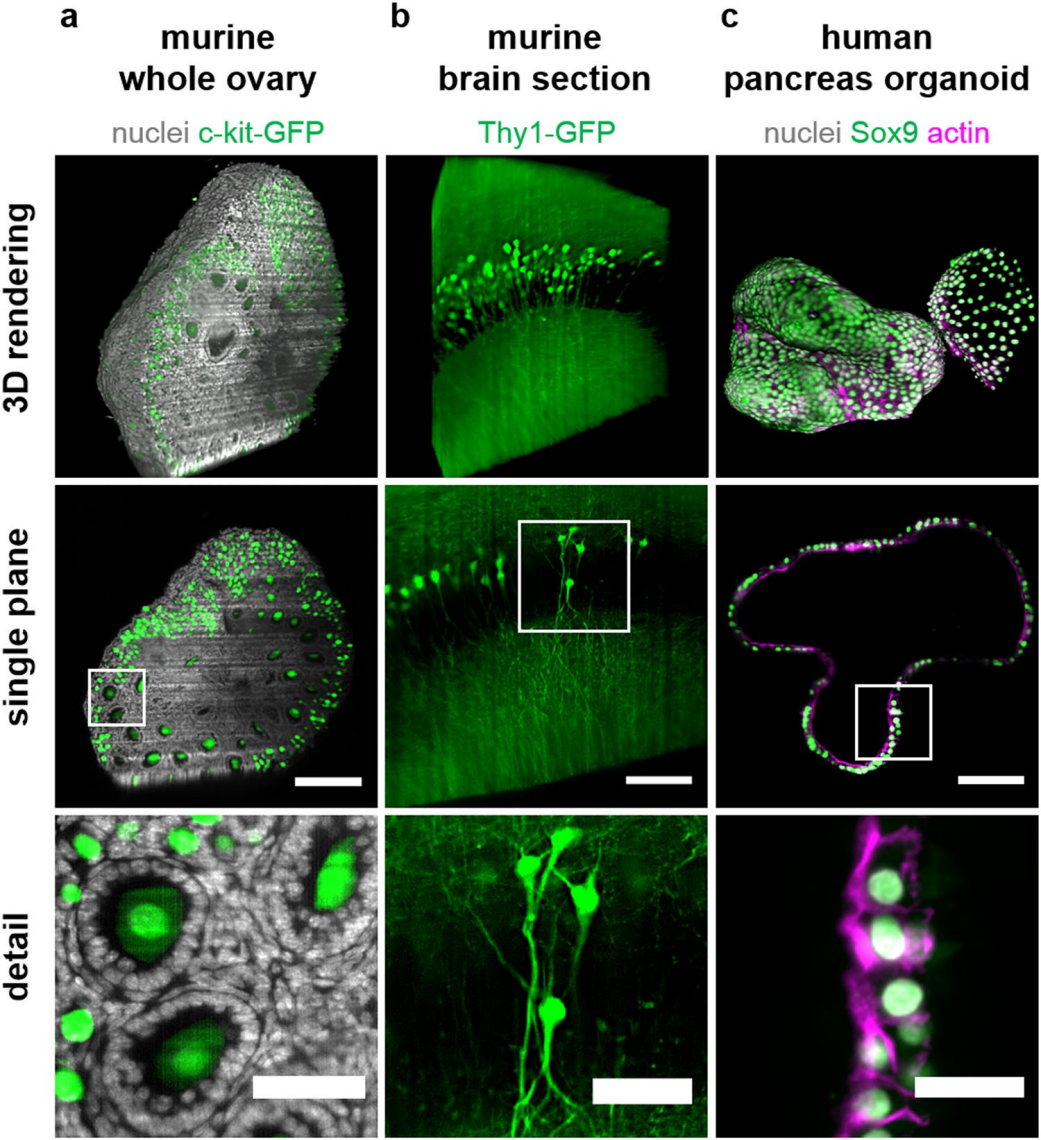
Specimens with heterogeneous optical densities mounted into ultra-thin FEP-foil cuvettes provide a high resolution at the subcellular level. Renderings, single central planes and detail views of: (**a**) A whole murine ovary of an eight day-old transgenic p-18 GFP-c-Kit mouse, in which all oocytes express GFP-c-Kit (green). Nuclei were stained with DAPI (grey). Clearly visible are primordial and primary oocytes, as well as the nuclei of surrounding, e.g. granulosa, cells. (**b**) A thick (diameter 1.5 mm) murine brain section of a mouse expressing GFP under the control of the Thy-1 promoter, e.g. in the granular layer within the dentate gyrus and (**c**) a native human pancreas organoid immuno-stained against the pancreas progenitor cell marker Sox9 (green), stained with phalloidin (magenta) and DAPI (grey). Specimens of a and b were optically cleared with CUBIC2, the specimen of c was not optically cleared. All samples were imaged with LSFM in ultra-thin FEP-foil cuvettes. Microscope: mDSLM. Objective lenses: Epiplan-Neofluar 2.5x/0.06 (excitation). N-Achroplan 10x/0.3 (detection). Excitation wavelength: 488 nm. Bandpass detection filter: 525/50 nm. Scale bar: 100 µm, detail view scale bar: 50 µm.

Murine ovary: Explanted and *ex vivo* cultured ovaries, expressing GFP-cKit specifically in all oocytes, were treated as previously published^46,47^. Staining was performed as published by Smyrek *et al*.^35^ with slight modifications. Briefly, ovaries were harvested from eight day-old (P8) female GFP-cKit mice and fixed in 4% PFA in PBS overnight. The ovaries were permeabilised with 0.3% Triton X-100 in PBS for 30 minutes at room temperature in a 96-well flat-bottom plate (Greiner), while shaking at 450 rpm. Next, the ovaries were treated with blocking buffer (0.3% Triton X-100, 0.05% Tween-20, 0.1% BSA and 10% donkey serum in PBS) for 2 hours at room temperature. After blocking, the ovaries were incubated with DAPI (Thermo Fisher; 1 μg/ml in blocking buffer) at 37 °C in a humidified incubator in the dark for 24 hours. Afterwards, the ovaries were washed three times with PBS in the dark for 20 minutes, and were kept in PBS at 4 °C, protected from light in a humidified incubator. The ovaries were washed four times in CUBIC2 solution before being transferred into the octagonal FEP-cuvettes containing CUBIC2 solution with forceps (Fig. 2c,d). To avoid floating ovaries, it is important to make sure that no air bubbles enter the cuvette.

**Figure 3:**
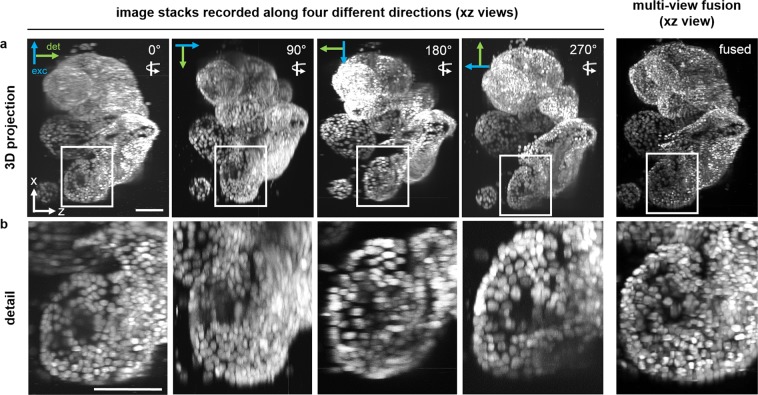
Projections and fusion of image stacks of human liver organoids recorded along four directions for multiple-view fusion. A cluster of human liver organoids in an ultra-thin square cross section FEP-foil cuvette. Four stacks consisting of 301 images each were recorded with a DSLM along four different directions, i.e. rotation angles around the y-axis of 90°, 180° and 270°. Shown are four 3D maximum intensity xz-projections that provide views of the specimen “from above”, i.e. along the y-axis. The projections for each rotation angle are aligned to show the specimen along a comparable orientation. Arrows indicate the orientations of the light paths of excitation (blue) and detection (green) for each angle. (**a**) Overviews of the entire organoids cluster. (**b**) Detailed views of one organoid in the cluster. Cell nuclei (DAPI). Microscope: mDSLM. Objective lenses: Epiplan-Neofluar 2.5x/0.06 (excitation). N-Achroplan 10x/0.3 (detection). Excitation wavelength: 405 nm. Bandpass detection filter: 447/25 nm. Scale bars: 100 µm.

Human and murine organoids: Human adult liver-derived organoids were initiated from biopsies of healthy donor liver (~0.5 cm^3^) collected during liver transplantation performed at the Erasmus MC – University Medical Center Rotterdam (NL). Liver-derived organoids were cultured in expansion medium for seven days as previously described^48^. Murine and human adult pancreas-derived organoids were obtained from Meritxell Huch (Gurdon Institute, Cambridge, UK). Pancreas-derived organoids were cultured as previously described^18^ with adjustments for human pancreas-derived organoids (Meritxell Huch, personal communication, unpublished data). Isolation of organoids from the embedding matrix (human liver organoids: Matrigel, Corning; human pancreas organoids: Cultrex BME2, Amsbio) for fixation and whole-mount staining was performed with slight modifications of protocols published by Broutier *et al*.^49,50^. Briefly, organoids were extracted from the matrix by washing three times with ice-cold 0.1% BSA in PBS. Organoids were fixed with 2% PFA in PBS on ice for 30 min. Whole-mount staining of the specimens was done using an adapted version of a protocol previously published by Smyrek *et al*.^35^. Human pancreas organoids were additionally immuno-stained against Sox9 (primary antibody: Merck Millipore, AB5535; secondary antibody: goat anti-rabbit IgG (H + L) Alexa Fluor 488, Thermo Fisher, A11008) and labelled with Alexa Fluor 568 Phalloidin (Thermo Fisher, 1:200). All the organoids were counter-stained with DAPI (Thermo Fisher; 1 μg/ml in PBS). The stained specimens were washed three times and stored in PBS at 4 °C. Liver organoid clusters (Fig. 3), still partially embedded in Matrigel, were gently transferred from an embryo dish (Fig. 1e) into ultra-thin FEP-foil cuvettes using a pipette with a 100 µl tip cut at the end in order to avoid damaging of the specimen due to shear forces (Fig. 1c,f). These cuvettes with a square cross section were attached to stainless steel pins and pre-filled with PBS. PBS was used as both mounting (in the cuvette) and imaging medium (in the DSLM chamber, Fig. 1g). The same mounting procedure was used for single human pancreas organoids (Fig. 2c). For nuclei segmentation, murine pancreas organoids were grown within ultra-thin FEP-foil cuvettes for three days (Supplementary Fig. 6). Prior to organoid seeding, the ultra-thin FEP-foil cuvettes were sterilised in 75% ethanol for at least 3 hours, washed thoroughly with PBS and dried. They were then filled with 20 µl of Matrigel containing organoid fragments, placed into a 48-well plate and fully covered with expansion medium. After three days of culture, the ultra-thin FEP-foil cuvettes containing the organoids embedded in Matrigel were briefly washed with PBS and fixed with 4% PFA in PBS supplemented with 1% glutaraldehyde (Electron Microscopy Sciences, 16020) for 30 minutes at 4 °C. After two short washing steps with PBS, up to three ultra-thin FEP-foil cuvettes were pooled in a 1.5 ml tube and stained with DAPI (Thermo Fisher; 1 μg/ml in PBS) overnight. For imaging, the filled FEP-cuvettes were attached to stainless steel pins using instant glue. PBS was used as imaging medium to fill the DSLM chamber.

#### Live specimens

**Figure 4:**
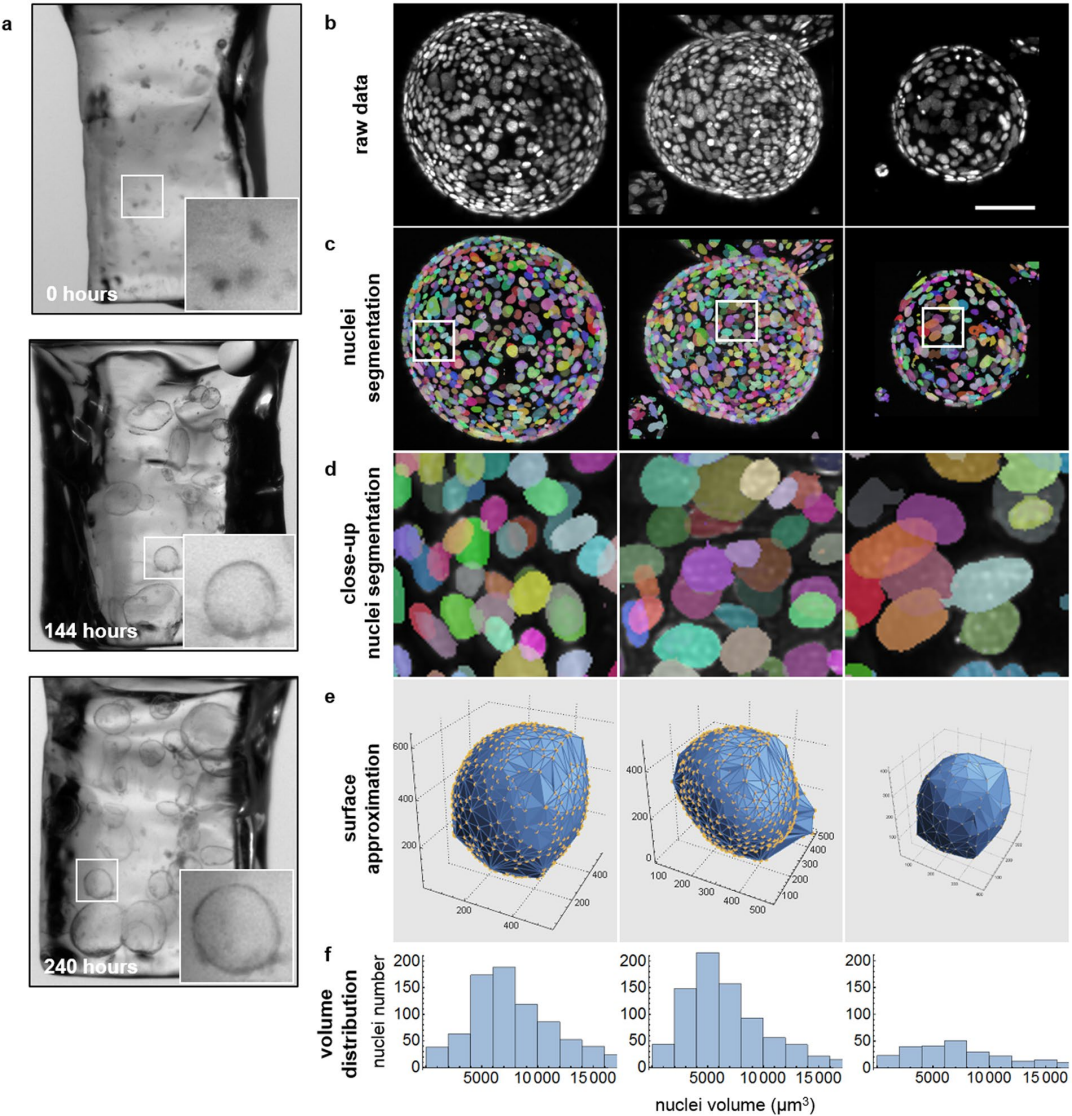
Long-term culture of murine pancreas organoids fixed and labelled inside ultra-thin FEP-foil cuvettes. (**a**) Organoids were directly seeded into ultra-thin FEP-foil cuvettes and cultured over a period of ten days (240 hours). The insets show healthy pancreas organoids 0, 144 and 240 hours after seeding. The organoids remain in the same cuvette during fixation, labelling and imaging. (**b**) Maximum intensity xz-projections of raw image stacks of three different pancreas organoids after fixation and DAPI staining inside the ultra-thin FEP-foil cuvette. Microscope: mDSLM. Objective lenses: Epiplan-Neofluar 2.5x/0.06 (excitation). N-Achroplan 10x/0.3 (detection). Excitation wavelength: 405 nm. Bandpass detection filter: 447/25 nm. Scale bar: 100 µm. (**c**) High image quality ensures a clear separation amongst the labeled nuclei, which is essential for semi-automated nuclei segmentation. Different colors indicate individual nuclei. (**d**) Close-ups of segmented nuclei. (**e**) The global morphology of the organoids is shown in surface approximations based on alpha shapes. (**f**) Histograms show the nuclei volume distribution within the three different organoids.

Live murine organoids in FEP-foil cuvettes: Murine pancreas organoids were also grown within ultra-thin FEP-foil cuvettes for up to 10 days. Overview images of the FEP-cuvettes containing living organoids were taken 0 h, 144 h and 240 h after seeding using the Zeiss SteREO Discovery.V8 equipped with a AxioCam ICc1 Camera (Fig. 4a).

#### Ethical approvals

Animal treatment of mice expressing GFP under the control of the Thy-1 promoter was performed under veterinary supervision in accordance with animal welfare regulations of the German animal protection law (Regierungspräsidium Darmstadt and Karlsruhe, Germany). All mice were maintained in an animal facility, and the experimental procedures were approved by the Animal Welfare office of the Regierungspräsidium Darmstadt and Karlsruhe. Animal care and handling of mice expressing GFP-cKit were performed according to the guidelines set by the World Health Organization (Geneva, Switzerland). The Ethics Approval REC No. 12/EE/0253 from the UK National Research Ethics Service (NRES) covers the ethical issues involved the generation and culture of the pancreas organoids used in the LSFM4LIFE project and in this work. Medical ethical approval for working with human liver organoids has been granted the Medical Ethical Committee (METC) of the Erasmus Medical Center in Rotterdam, The Netherlands (MEC-2014-060). Patients provided informed consent and all methods were performed in accordance with the relevant guidelines and regulations.

### Optical clearing

#### Whole murine ovaries and brain sections

For clearing of whole murine ovaries^46^ and large tissue sections of murine brain, we used CUBIC2 clearing solution^40^. Briefly, 50% w/v sucrose, 25% w/v urea and 15% w/v deionized water were mixed and stirred at 60 °C. After all components were dissolved, 10% w/v 2,2′,2′’-nitrilotriethanol was added and stirred at room temperature. The clearing solution was de-gassed by placing it into a desiccator for about 20 minutes. Finally, the refractive index was determined (n = 1.49). The specimens were immersed in CUBIC2 in a reaction tube overnight.

#### Murine kidney

Optical clearing of murine kidney was performed with ethyl cinnamate (ECi, n = 1.58)^27^. The specimen was dehydrated with increasing concentrations of ethanol in ultrapure water. After the last dehydration step with 100% ethanol, the specimen was immersed in ECi in a reaction tube overnight.

### Cleaning procedure for re-using the ultra-thin FEP-foil cuvettes

The imaged specimen was removed and the cuvettes were transferred into a 15 ml reaction tube. Next, the cuvettes were washed in a 1% solution of Hellmanex-II in ultrapure water at room temperature on a rotator overnight. After washing, the Hellmanex-II (Hellma Analytics, Müllheim, DE) solution was discarded and the cuvettes were rinsed twice with ultrapure water for at least one hour on the rotator. After drying at 60 °C for at least one hour, the cuvettes can be re-used. Slight deformations of the cuvette’s walls caused by the washing procedure can be corrected by gentle stretching with of a pair of tweezers.

### LSFM imaging

The FEP-cuvettes were glued onto stainless steel holders (Fig. 1f) and images were acquired with one custom-built monolithic digital-scanned light sheet-based fluorescence microscope (mDSLM)^11^, which features a motorized xyzϑ-stage placed below the specimen chamber (Fig. 1g). The microscope was equipped with an Epiplan-Neofluar 2.5x/0.06 illumination objective (Carl Zeiss), an N-Achroplan 10x/0.3 detection objective (Carl Zeiss) and a Clara CCD camera (ANDOR Technology, Ireland). Laser wavelength and bandpass filter sets (centre wavelength/full-width at half maximum): 561 nm, 607/70 nm; 488 nm; 525/50 nm, 405 nm, 447/50 nm.

### Imaging of sub-wavelength fluorescent beads

The fluorescence intensities of single TetraSpeck microspheres (Thermo Fisher) were used as a measure for the relative axial (yz) and lateral (xy) fluorescence intensity distributions in PBS and in CUBIC2 microscopy immersion media, respectively. Microspheres were embedded with low-melting point agarose in square cross section ultra-thin FEP-foil cuvettes. We used an mDSLM equipped with an Epiplan-Neofluar 2.5x/0.06 illumination objective (Carl Zeiss) and an N-Achroplan 10x/0.3 detection objective (Carl Zeiss). The microspheres were excited at 488 nm. A bandpass filter centred at 525/50 was used to record the fluorescence emitted by the beads. Images of microbeads close to centre of the agarose column were recorded and analysed with Fiji (ImageJ version 1.51d, Java version 1.6.0_24). Line intensity profiles were measured by using the line selection tool of Fiji and the Analyze/Plot Profile tool. The image of each microsphere was normalized by the maximal intensity value. The intensity profiles of 10 fluorescent microbeads along the xy- and yz-direction were analysed for each condition. After a Gaussian fitting of the lateral and axial distributions, the full width half maxima (FWHM) were determined as a measure for the resolution^51^.

### Image processing

Raw image stacks were pre-processed with Fiji (ImageJ version 1.51d, Java version 1.6.0_24)^42^. The stacks were cropped to the region of interest. The background intensity was subtracted from every slice using the function Subtract Background (Fiji, ball radius of 60 pixels). The stacks were resliced with a factor of four along the z-axis. 3D maximum intensity projections were generated with Fiji (projection method: brightest point, 360° rotation angle, angular increments were set at 1° or 10°). The multi-view registration and fusion of the organoid stack displayed in Raw image stacks were pre-processed with Fiji (ImageJ version 1.51d, Java version 1.6.0_24)^42^. The stacks were cropped to the region of interest. The background intensity was subtracted from every slice using the function Subtract Background (Fiji, ball radius of 60 pixels). The stacks were resliced with a factor of four along the z-axis. 3D maximum intensity projections were generated with Fiji (projection method: brightest point, 360° rotation angle, angular increments were set at 1° or 10°). The multi-view registration and fusion of the organoid stack displayed in Fig. 3 was performed with Huygens Essential SPIM/Light Sheet Fusion & Deconvolution Wizard (Scientific Volume Imaging, NL)^52^. The volume rendering of the ovary in Fig. 2 was performed with the Fiji plugin 3D viewer^53^. Stripe filtering and deconvolution of the murine kidney dataset shown in Supplementary Fig. 4 were conducted with the Fiji plugins Parallel Iterative Deconvolution 3D v1.12^54^ and with the VSNR V2 Variational Stationary Noise Remover^55^, respectively. In the VSNR plugin, a Gabor filter with a 90° angle, sigma_x = 3, sigma_y = 100, Lambda = 0 and a noise level = 0.3 were selected. The stack was processed in Mode 2D.

### Automated nuclei segmentation

Nuclei segmentation was performed as previously published in Schmitz *et al*. 2017^43^. Raw image stacks of the nuclei channel were pre-processed in Fiji (ImageJ version 1.51 h, Java version 1.8.0_66). The raw image stacks were cropped to the region of interest. Cell nuclei were extracted by means of an automated three-dimensional segmentation pipeline implemented in Wolfram Mathematica (version 10.3). In brief, pre-processed image stacks were rescaled with a factor of four along the z-axis to obtain isotropic voxels. Hence, in the resized 3D-image stacks, the voxels are isotropic with a pitch of 0.654 µm. The images were then deconvolved with a median filter range of three pixels. Cell nuclei segmentation was achieved by local thresholding and a marker-controlled immersion-based watershed algorithm. The local threshold corresponds to the mean of the local intensity distribution in a range of ten voxels. Marker points for the watershed algorithm were detected by a multiscale Laplacian of Gaussian seed detector with a minimal range of eight and a maximum range of twelve voxels^56,57^. Marker points were increased by morphological dilation with a spherical structuring element and a radius of three voxel. Using the marker points as starting points, the final segmentation result was obtained by an immersion-based watershed algorithm^58^. Using Edelsbrunner’s algorithm^59^, the cell nuclei centroids were used to compute an alpha shape with the alpha parameter set to 240 voxels. From the alpha shape, the approximate spheroid volume could be readily obtained. A cell graph^60,61^ was constructed with an edge distance threshold of 60 voxels capturing the spatial arrangement of cells and the local cell density within an organoid. The maximal outlier distance was 55 voxels. Features of all cell nuclei that had a volume greater than 500 and smaller than 18000 voxels, and global features of the spheroid were stored in tabular format.

## Results

### Fabrication of ultra-thin FEP-foil cuvettes can be easily adapted to specimen type and size

We designed two positive 3D-printable moulds for the fabrication of ultra-thin FEP-foil cuvettes (Fig. 1a). Cuvettes with a square cross section are suitable for embedding small specimens in a diameter range of micro- to millimetres. Cuvettes with an octagonal cross section are used with specimens in a diameter range of several milli- to centimetres. The vacuum forming fabrication process results in a durable yet flexible shape. Also, the resulting stretching of the original FEP-foil with a thickness of 50 µm by a factor between 4 and 6 provides ultra-thin FEP-foils with a thickness of between 10 µm and 12 µm (Supplementary Fig. 2). Thinner original FEP-foils with a thickness of 25 µm did not provide mechanically stable extruded foils. Single cuvettes were cut from the array (Fig. 1b, right) and glued onto metal pins (sample holders) before depositing the specimen together with mounting medium into the cuvettes (Fig. 1f). Since these ultra-thin FEP-foil cuvettes are both stable and resilient, small and large specimens such as dense organoid clusters (Fig. 1e) and thick murine brain sections (Fig. 1d) are easily deposited and positioned inside the cuvette using a forceps or pipettes (Fig. 1c). Finally, the ultra-thin FEP-foil cuvette/metal pin sample holders were placed into the LSFM sample chamber filled with the imaging medium (Fig. 1g).

### Ultra-thin FEP foils minimise image deterioration caused by refractive index mismatches

**Figure 5:**
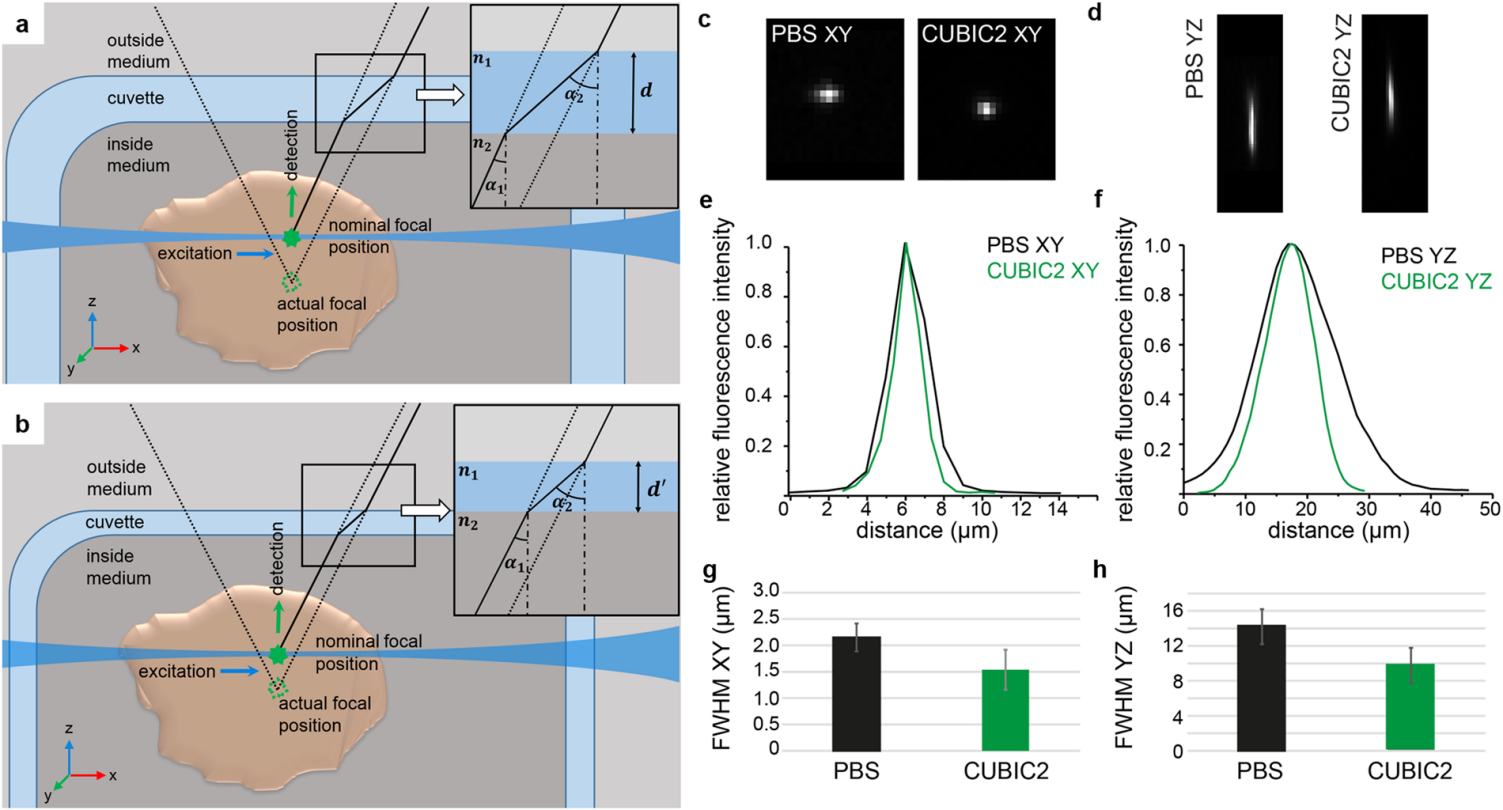
The reduced wall thickness of ultra-thin FEP-foil cuvettes minimises the spherical aberration caused by refractive index mismatches. Due to the refractive-index mismatch, the nominal focal position (NFP) is shifted with respect to the actual focal position (AFP). Thus, point spread function (PSF) and image are spherically aberrated. (**a**) A cleared specimen is immersed into a medium with a refractive index ${n}_{2}$ within an ultra-thin FEP-foil cuvette. The cuvette’s walls have a refractive index ${n}_{1}$ with ${n}_{2} > {n}_{1}$ and a thickness $d$. The cuvette is inserted into the LSFM chamber. Raytracing visualises the effects of refractive index mismatch, the thickness $d$ and the angular dispersion on the spherical aberration. Light emitted by a fluorophore in the specimen (green dot) with an angle of incidence ${\alpha }_{1}$ with respect to the wall (inset), has an angle of refraction ${{\boldsymbol{\alpha }}}_{{\bf{2}}}$. (**b**) A smaller thickness $d^{\prime}  < d$ reduces the distance between the nominal focal position NFP and AFP, minimising the spherical aberration. Identical consideration are valid for the illumination pathway (not shown in the drawing). However, both the very thin foil (12.4 µm ± 1.6) and the low numerical aperture of the illuminating beam result in a much smaller shift compared to the one in the detection pathway (see also Hell *et al*.^63^ for a systematic analysis of the aberration due to refractive index mismatches). (**c**–**h**) Comparison of the lateral and axial extents of fluorescent microspheres imaged within ultra-thin FEP-foil cuvettes in PBS and CUBIC2. (**c**,**d**) Images of the fluorescent microspheres in xy and yz planes, respectively. Lateral and axial fluorescence intensity profiles of two representative microspheres (diameter 0.5 µm) at 488 nm in PBS and CUBIC2. Microscope: mDSLM. Objective lenses: Epiplan-Neofluar 2.5x/0.06 (excitation). N-Achroplan 10x/0.3 (detection). Excitation wavelength: 488 nm. Bandpass detection filter: 525/50 nm. (**e**,**f**) Fluorescence intensity profiles of two representative microspheres. (**g**,**h**) Comparison of the full width half maximum (FWHM) values in PBS and CUBIC2 along a lateral and the axial directions. Error bars show the standard deviation (n = 10). The FWHM was used as a relative measure for the PSF.

Figure 5a,b illustrate the spherical aberration induced by the refractive index mismatch and the wall thickness. The ultra-thin foil produced during the vacuum-forming process reduces the wall thickness and minimises the image deterioration caused by the refractive index mismatch. To quantify the imaging properties of the ultra-thin FEP-foil cuvettes, we imaged sub-wavelength fluorescent microsphere inside the FEP-cuvettes with an mDSLM in different water-based media and measured the full width-half maximum of their lateral and axial intensity profile. The microspheres were embedded in PBS/agarose gel inside the cuvettes, which were subsequently immersed in the DSLM sample chamber filled with either PBS or CUBIC2^40^. Images of the lateral (xy) and axial (yz) extents of the fluorescent microspheres in PBS and CUBIC2 are shown in Fig. 5c,d, respectively. Figure 5e,f display the corresponding lateral and axial extents of the bead images in PBS and CUBIC2. Despite the sub-optimal refractive index matching for the CUBIC2 setup, in which the emitted light has to pass through the PBS/agarose gel inside the cuvette, FEP (n = 1.34), and CUBIC2 (n = 1.48), the full width half maxima (FWHM) of the lateral and axial extents in CUBIC2 are smaller (30%) than the ones in PBS (Fig. 5g,h and Supplementary Table 1).

### Ultra-thin FEP-foil cuvettes are generally applicable to *in toto* imaging

In order to test the applicability of ultra-thin FEP-foil cuvettes, we imaged a variety of biologically diverse specimens applying different sample mounting techniques. The specimens included CUBIC2-optically cleared whole murine ovaries, thick murine brain sections, ethyl cinnamate (ECi)-cleared murine kidney sections and native (non-cleared) human pancreas organoids (Fig. 2). Individual whole murine ovaries expressing GFP-c-kit were stained with DAPI, cleared with CUBIC2 and placed into ultra-thin FEP-foil cuvettes with a square cross section (see Movie 1). It was possible to image the complete densely-packed organ with a 10x/0.3 objective lens, i.e. post-acquisition image stitching was not required. We easily distinguished the stromal cells, the boundary of the *teca*, the cell layers in the *granulosa* and the cytoplasm of the oocytes (Fig. 2a, detail view). Supplementary Figure 3 shows 25 optical sections through the whole ovary of an eight day-old (P8) mouse. We also achieved promising results with thick murine brain sections, in which GFP was expressed under the control of the Thy-1 promoter (see Movie 2). Figure 2b (single plane, detail view) shows an optical section of the granular layer in the dentate gyrus of the murine hippocampus. The cleared brain slice with a size of 5 mm × 5 mm was inserted into an ultra-thin FEP-foil cuvette with an octagonal cross section. A suitable region of interest (ROI) was selected and imaged with a DSLM. A maximum intensity projection of a Z-stack composed of 101 slices, a three-dimensional surface rendering, and a detail view show that the image quality is well-suited for morphological investigations of lipid-rich tissues. The image shows a single ROI and no subsequent image stitching was performed. The results of ovary and brain imaging demonstrate that the combination of clearing with CUBIC2 and ultra-thin FEP-foil cuvettes allows *in toto* analyses of mammalian organs and thick tissue slices. In order to determine the performance with organic clearing agents with a refractive index of around 1.5, we cleared a murine kidney section with ethyl cinnamate (ECi). The auto-fluorescence of the tissue was excited at 488 nm (see Supplementary Movie 1). The image quality is not deteriorated, despite the large refractive index mismatch between the FEP-foil (n = 1.34) and ECi (n = 1.56) (Supplementary Fig. 4). We further tested the performance of the ultra-thin FEP-foil cuvettes in combination with immuno-labelled organoids (see Movie 3). Human pancreas organoids were stained with DAPI and phalloidin to visualise the nuclei and actin cytoskeleton, respectively, as well as immuno-stained against the pancreas progenitor marker Sox9 (Fig. 2c). The close-up provides a detailed overview of the spatial distribution of the actin cytoskeleton, which is most prominent at the membranes facing the organoid lumen. In summary, these data suggest that the ultra-thin FEP-foil cuvettes are applicable to a wide variety of biologically diverse specimens, including native (non-cleared) and optically cleared specimens. They allow the microscopic assessment of subcellular structures in large specimens.

### Ultra-thin FEP-foil cuvettes are suitable for multiple-view imaging and three-dimensional multiple-view reconstruction

Delicate multi-cellular structures such as organoids, constituted by a cell-monolayer and a hollow lumen, are not suitable for optical clearing. The clearing procedures result in damage of the original architecture and their collapse. Thus, multiple-view imaging is required to achieve high-quality and isotropic 3D image stacks of the organoids. We imaged a dense cluster of several human liver organoids partially embedded in Matrigel and stained with DAPI inside ultra-thin FEP-foil cuvettes with a square cross section. Image stacks were recorded along four directions (0°, 90°, 180°, 270°) (Fig. 3). This cluster (Supplementary Figure 5), is a complex and highly light-scattering specimen. The small size of the square cross section FEP-cuvette (side length: 2 mm) allows for an unobstructed observation. Maximum intensity xz-projections along four different directions are presented in Fig. 3a. Each projection was aligned with the 0° projection. The detail views of the organoid cluster (Fig. 3b) reveal optical distortions that commonly occur with highly scattering samples. The elongated shape of the nuclei present in each single-view projection (particularly evident in Fig. 3b), depends on the worse optical resolution along the zx axis compared to the xy axis (due to the more elongated PSF along xz). In contrast, the dataset resulting from the fusion of the four single views shows an isotropic resolution along at the xy, xz, and yz planes (Fig. 3, panels “multiple-view fusion”). Multiple-view imaging and multiple-view fusion reduce the optical distortions and provide more detailed information on the subcellular level (Fig. 3b, Movie 4). These data indicate the improvement of the image quality resulting from multiple-view imaging with ultra-thin FEP-foil cuvettes and multiple-view fusion of native (non-cleared) specimens.

### Ultra-thin FEP-foil cuvettes are suitable for long-term culture of organoids and high quality imaging for quantitative image-based analyses

Image-based analyses require a good signal-to noise ratio and a sufficient spatial resolution. We cultured organoids embedded in matrix directly inside the ultra-thin FEP-foil cuvettes in order to preserve their morphology during fixation, staining and imaging (Supplementary Fig. 6). Murine pancreas organoid fragments were seeded in ultra-thin FEP-foil cuvettes and covered with the expansion medium in culture dishes. The organoids showed unhampered growth for up to ten days within the FEP cuvettes (Fig. 4a), demonstrating that direct culture of the organoids in the FEP cuvettes is feasible. Subsequently, the organoid cultures were fixed and stained with DAPI within the ultra-thin FEP-foil cuvettes. We applied our recently developed multi-scale image analysis pipeline^43^ to process the acquired image data. The cell nuclei (Fig. 4b) were segmented (Fig. 4c) and basic morphological features were extracted for individual organoids (see Movie 5). In the middle column of Fig. 4b, a few nuclei of a neighbouring organoid can be observed that were also segmented. Figure 4d shows detailed views of segmented nuclei, indicating a good detection performance due to high image quality. Based on the nuclei segmentation data, surface areas and volumes of the organoids were approximated in 3D (Fig. 4e, Supplementary Table 2). Furthermore, nuclei volume distributions within individual organoids were determined, which reveals differing median nuclei volumes for each organoid (Fig. 4f).

## Discussion

The pressure to work with delicate 3D cell cultures such as tissue sections, organoids and spheroids continues to rise, hence the demand for simple but versatile sample preparation methods for LSFM. In order to provide high quality imaging properties, we developed ultra-thin FEP-foil chambers, in particular cuvettes, which are easily manufactured and adapted to a sample’s properties. We describe the fabrication of ultra-thin FEP-foil cuvettes as specimen holders for LSFM. We also demonstrate their applicability with representative 3D image stacks of structurally intact native and optically cleared whole organs, thick tissue sections, single organoids and dense organoid clusters.

In the pioneering days of LSFM, we introduced FEP-foil sample holders to study the 3D dynamic instability of 3D microtubule asters^37^. These early FEP-foil holders were produced by wrapping the FEP-foil sheet around a glass capillary and gluing it along the capillary’s axis to form a cylindrical container. Although we obtained good image quality using FEP-cylinders, the technique held several drawbacks, including leakage and restricted rotation for multiple-views imaging due to light scattering caused by the glue line. The ultra-thin FEP-foil cuvettes overcome these issues.

The production of ultra-thin FEP-cuvettes combines 3D printing and vacuum forming. Both technologies are affordable and easily available. They provide microscope developers and users with a lot of freedom for customisation. Using the newly developed flexible, resilient and seamless ultra-thin FEP-foil cuvettes as sample containers for LSFM simplifies sample preparation and handling of large specimens. In particular, the positioning of the specimen inside the cuvettes is much easier compared to rigid and fragile cuvette-like sample holders such as glass capillaries^35,36^. Another advantage of the new ultra-thin FEP-foil cuvettes is their compatibility with broad ranges of native and optically cleared biological specimens, mounting media and refractive indices. We verified a high tolerance against spherical aberration originating from refractive index mismatches with frequently used optical clearing solutions such as CUBIC2 (n = 1.49) and ECi (n = 1.58). This tolerance is based on the very thin wall of the cuvette (10 µm–12 µm), which results from stretching the heated FEP-foil (original thickness about 50 µm) over the positive moulds in the vacuum forming process. The foil stretch is quantified by the *draw ratio* (=surface area/footprint). The calculated draw ratio, based on the geometry outlined in Supplementary Figure 2d, is 6. The measured draw ratio obtained from the average footprint and surface areas is around 4 (Supplementary Fig. 2d, table). The draw ratio depends on the process of vacuum forming: the temperature of the foil, the suction pressure, the shape of the mould, the ratios of the mould’s geometry, the curvature of the edges and the mould’s material (Supplementary Fig. 2d).

An important property is that both the shape and the seamless nature of ultra-thin FEP-foil cuvettes allow for an unrestricted rotation of the sample for multiple-view imaging. This simplifies the 3D reconstruction of multiple-views data sets, which improves the image quality of highly scattering specimens. Furthermore, the image quality is suitable for the application of state-of-the-art multiple-scale image analysis tools^43^ and, thus, meets the requirements of quantitative methods for system-based analysis approaches. Beyond what we demonstrated, ultra-thin FEP-foil cuvettes are also suitable for long-term live imaging, since temperature and gas exchange remain controllable. FEP is an inert and bio-compatible material that does not interfere with the normal cell physiology^62^. Since FEP is impermeable to liquids, the composition of the internal media is under control since no external contamination or dilution due osmosis occurs. E,g, we cultured pancreas organoids embedded in Matrigel inside the ultra-thin FEP-foil cuvette for over a week prior to imaging. Moreover, we conducted live imaging to study the growth and morphogenesis of pancreas organoids for over 120 hours inside a FEP-cuvette (*Lotta Hof, Till Moreth, unpublished data*).

Besides FEP, other thermoplastic polymeric foils with a broad range of refractive indices are available on the market (Supplementary Table 3). Our preliminary tests of cyclo-olefine polymer foil (ZEONEX) with a thickness of 40 µm and a refractive index of 1.52, i.e. close to organic clearing solutions, has been performed successfully. The production of specimen holders from these foils by vacuum forming will further extend the usefulness of our approach.

In summary, ultra-thin FEP-foil cuvettes are “universal” and “congruent” sample holders that greatly simplify and standardise imaging of a broad spectrum of specimens, from small to large, both native and optically cleared. Introducing increasingly user-friendly and efficient procedures for specimen mounting in LSFM will encourage even more life scientists to take advantage of the most useful microscopy to address and answer biological questions.

## Supplementary information

Optically cleared murine ovary

Optically cleared mouse hippocampus

Human pancreas organoid

Human liver organoids

Segmented nuclei in a murine pancreas organoid

Mouse kidney optically cleared with ethyl cinnamate

Supplementary information

## References

[1] Pampaloni F, Chang BJ, Stelzer EHK (2015). Light sheet-based fluorescence microscopy (LSFM) for the quantitative imaging of cells and tissues. Cell Tissue Res..

[2] Pampaloni F, Ansari N, Stelzer EHK (2013). High-resolution deep imaging of live cellular spheroids with light-sheet-based fluorescence microscopy. Cell and Tissue Research.

[3] Andilla J (2017). Imaging tissue-mimic with light sheet microscopy: A comparative guideline. Sci. Rep..

[4] Rios AC, Clevers H (2018). Imaging organoids: A bright future ahead. Nat. Methods.

[5] Siedentopf H, Zsigmondy R (1902). Uber Sichtbarmachung und Größenbestimmung ultramikoskopischer Teilchen, mit besonderer Anwendung auf Goldrubingläser. Ann. Phys..

[6] Voie AH, Burns DH, Spelman FA (1993). Orthogonal-plane fluorescence optical sectioning: Three-dimensional imaging of macroscopic biological specimens. J. Microsc..

[7] Huisken J, Swoger J, Bene FD, Wittbrodt J, Stelzer EHK (2004). Live Embryos by Selective Plane Illumination Microscopy. Science (80-.)..

[8] Verveer PJ (2007). High-resolution three-dimensional imaging of large specimens with light sheet–based microscopy. Nat. Methods.

[9] Engelbrecht CJ, Stelzer EH (2006). Resolution enhancement in a light-sheet-based microscope (SPIM). Opt. Lett..

[10] Dodt HU (2007). Ultramicroscopy: Three-dimensional visualization of neuronal networks in the whole mouse brain. Nat. Methods.

[11] Keller PJ, Schmidt AD, Wittbrodt J, Stelzer EHK (2008). Reconstruction of zebrafish early embryonic development by scanned light sheet microscopy. Science (80-.)..

[12] Chen, B. C. *et al*. Lattice light-sheet microscopy: Imaging molecules to embryos at high spatiotemporal resolution. *Science (80-.)*. **346** (2014). 10.1126/science.1257998 PMC4336192 25342811

[13] Wan Y, McDole K, Keller PJ (2019). Light-Sheet Microscopy and Its Potential for Understanding Developmental Processes. Annu. Rev. Cell Dev. Biol..

[14] Glaser, A. K. *et al*. Light-sheet microscopy for slide-free non-destructive pathology of large clinical specimens. *Nat. Biomed. Eng*. **1** (2017). 10.1038/s41551-017-0084 PMC5940348 29750130

[15] Corsetti S, Gunn-Moore F, Dholakia K (2019). Light sheet fluorescence microscopy for neuroscience. J. Neurosci. Methods.

[16] Huisken J, Swoger J, Del Bene F, Wittbrodt J, Stelzer EHK (2004). Optical sectioning deep inside live embryos by selective plane illumination microscopy. Science (80-.)..

[17] Strobl F, Schmitz A, Stelzer EHK (2015). Live imaging of Tribolium castaneum embryonic development using light-sheet–based fluorescence microscopy. Nat. Protoc..

[18] Huch M (2013). Unlimited *in vitro* expansion of adult bi-potent pancreas progenitors through the Lgr5/R-spondin axis. EMBO J..

[19] Pampaloni F, Stelzer EHK, Leicht S, Marcello M (2010). Madin-Darby canine kidney cells are increased in aerobic glycolysis when cultured on flat and stiff collagen-coated surfaces rather than in physiological 3-D cultures. Proteomics.

[20] Pampaloni F (2014). Tissue-culture light sheet fluorescence microscopy (TC-LSFM) allows long-term imaging of three-dimensional cell cultures under controlled conditions. Integr. Biol. (United Kingdom).

[21] Desmaison A, Lorenzo C, Rouquette J, Ducommun B, Lobjois V (2013). A versatile sample holder for single plane illumination microscopy. J. Microsc..

[22] Spalteholz, W. Über das Durchsichtigmachen von menschlichen und tierischen Präparaten und seine theoretischen Bedingungen, nebst Anhang: Über Knochenfärbung. (S. Hirzel, 1914).

[23] Dent Ja, Polson aG, Klymkowsky MW (1989). A whole-mount immunocytochemical analysis of the expression of the intermediate filament protein vimentin in Xenopus. Development.

[24] Höckendorf B, Lavis LD, Keller PJ (2014). Making biology transparent. Nat. Biotechnol..

[25] Richardson DS, Lichtman JW (2015). Clarifying Tissue Clearing. Cell.

[26] Pan C (2016). Shrinkage-mediated imaging of entire organs and organisms using uDISCO. Nat. Methods.

[27] Klingberg A (2017). Fully Automated Evaluation of Total Glomerular Number and Capillary Tuft Size in Nephritic Kidneys Using Lightsheet Microscopy. J. Am. Soc. Nephrol..

[28] Tomer R, Ye L, Hsueh B, Deisseroth K (2014). Advanced CLARITY for rapid and high-resolution imaging of intact tissues. Nat. Protoc..

[29] Ke M-T, Fujimoto S, Imai T (2013). SeeDB: a simple and morphology-preserving optical clearing agent for neuronal circuit reconstruction. Nat. Neurosci..

[30] Tainaka K (2014). Whole-body imaging with single-cell resolution by tissue decolorization. Cell.

[31] Wan P (2018). Evaluation of seven optical clearing methods in mouse brain. Neurophotonics.

[32] Xu Jianyi, Ma Yilin, Yu Tingting, Zhu Dan (2018). Quantitative assessment of optical clearing methods in various intact mouse organs. Journal of Biophotonics.

[33] Abadie S (2018). 3D imaging of cleared human skin biopsies using light-sheet microscopy: A new way to visualize in-depth skin structure. Ski. Res. Technol..

[34] Stefaniuk M (2016). Light-sheet microscopy imaging of a whole cleared rat brain with Thy1-GFP transgene. Sci. Rep..

[35] Smyrek I, Stelzer EHK (2017). Quantitative three-dimensional evaluation of immunofluorescence staining for large whole mount spheroids with light sheet microscopy. Biomed. Opt. Express.

[36] Wenzel C (2014). 3D high-content screening for the identification of compounds that target cells in dormant tumor spheroid regions. Exp. Cell Res..

[37] Keller PJ, Pampaloni F, Stelzer EHK (2007). Three-dimensional preparation and imaging reveal intrinsic microtubule properties. Nat. Methods.

[38] Kaufmann A, Mickoleit M, Weber M, Huisken J (2012). Multilayer mounting enables long-term imaging of zebrafish development in a light sheet microscope. Development.

[39] Boothe T (2017). A tunable refractive index matching medium for live imaging cells, tissues and model organisms. Elife.

[40] Susaki EA (2014). Whole-brain imaging with single-cell resolution using chemical cocktails and computational analysis. Cell.

[41] Staudt T, Lang MC, Medda R, Engelhardt J, Hell SW (2007). 2,2′-Thiodiethanol: A new water soluble mounting medium for high resolution optical microscopy. Microsc. Res. Tech..

[42] Schindelin J (2012). Fiji: An open-source platform for biological-image analysis. Nat. Methods.

[43] Schmitz, A., Fischer, S. C., Mattheyer, C., Pampaloni, F. & Stelzer, E. H. K. Multiscale image analysis reveals structural heterogeneity of the cell microenvironment in homotypic spheroids. *Sci. Rep*. **7**, 43693 (1–13) (2017). 10.1038/srep43693 PMC5334646 28255161

[44] Feng G (2000). Imaging neuronal subsets in transgenic mice expressing multiple spectral variants of GFP. Neuron.

[45] Stefani J (2018). Disruption of the microglial ADP receptor P2Y13 enhances adult hippocampal neurogenesis. Front. Cell. Neurosci..

[46] Tuppi Marcel, Kehrloesser Sebastian, Coutandin Daniel W., Rossi Valerio, Luh Laura M., Strubel Alexander, Hötte Katharina, Hoffmeister Meike, Schäfer Birgit, De Oliveira Tiago, Greten Florian, Stelzer Ernst H. K., Knapp Stefan, De Felici Massimo, Behrends Christian, Klinger Francesca Gioia, Dötsch Volker (2018). Oocyte DNA damage quality control requires consecutive interplay of CHK2 and CK1 to activate p63. Nature Structural & Molecular Biology.

[47] Rossi V (2017). LH prevents cisplatin-induced apoptosis in oocytes and preserves female fertility in mouse. Cell Death Differ..

[48] Huch M (2013). *In vitro* expansion of single Lgr5 + liver stem cells induced by Wnt-driven regeneration. Nature.

[49] Broutier L (2016). Culture and establishment of self-renewing human and mouse adult liver and pancreas 3D organoids and their genetic manipulation. Nat. Protoc..

[50] Broutier L (2017). Human primary liver cancer-derived organoid cultures for disease modeling and drug screening. Nat. Med..

[51] Stelzer EHK (1998). Contrast, resolution, pixelation, dynamic range and signal- to-noise ratio: fundamental limits to resolution in fluorescence light microscopy. J. Microsc..

[52] Verveer PJ (2018). Restoration of Light Sheet Multi-View Data with the Huygens Fusion and Deconvolution Wizard. Micros. Today.

[53] Schmid B, Schindelin J, Cardona A, Longair M, Heisenberg M (2010). A high-level 3D visualization API for Java and ImageJ. BMC Bioinformatics.

[54] Wendykier, P. High Performance Java Software for Image Processing. (James T. Laney School of Graduate St, 2009).

[55] Fehrenbach J, Weiss P, Lorenzo C (2012). Variational Algorithms to Remove Stationary Noise: Applications to Microscopy Imaging. IEEE Trans. Image Process..

[56] Lindeberg T (1998). Feature detection with automatic scale selection. Int. J. Comput. Vis..

[57] Stegmaier J (2014). Fast segmentation of stained nuclei in terabyte-scale, time resolved 3D microscopy image stacks. PLoS One.

[58] Vincent L, Soille P (1991). Watersheds in digital spaces: An efficient algorithm based on immersion simulations. IEEE Trans. Pattern Anal. Mach. Intell..

[59] Edelsbrunner H, Kirkpatrick D, Seidel R (1983). On the shape of a set of points in the plane. IEEE Trans. Inf. Theory.

[60] Gunduz C, Yener B, Gultekin SH (2004). The cell graphs of cancer. Bioinformatics.

[61] Schäfer, H. *et al*. CD30 cell graphs of Hodgkin lymphoma are not scale-free - An image analysis approach. *Bioinformatics*, 10.1093/bioinformatics/btv542 (2015). 10.1093/bioinformatics/btv542 26363177

[62] Chen M, Zamora PO, Som P, Peña LA, Osaki S (2003). Cell attachment and biocompatibility of polytetrafluoroethylene (PTFE) treated with glow-discharge plasma of mixed ammonia and oxygen. J. Biomater. Sci. Polym. Ed..

[63] Hell S, Reiner G, Cremer C, Stelzer EHK (1993). Aberrations in confocal fluorescence microscopy induced by mismatches in refractive index. J. Microsc..

