# Supplementary material for: Ultra-thin fluorocarbon foils optimise multiscale imaging of three-dimensional native and optically cleared specimens
Source: Sci Rep. 2019 Nov 21;9:17292. doi: 10.1038/s41598-019-53380-2 (PMC6872575; doi:10.1038/s41598-019-53380-2)
Supplement: Supplementary file 7 — Supplementary information [file 41598_2019_53380_MOESM7_ESM.pdf]

Ultra-thin fluorocarbon foils optimise multiscale imaging of three-dimensional native and optically cleared specimens

Katharina Hötte[1], Michael Koch[1], Lotta Hof[1], Marcel Tuppi[2], Till Moreth[1], Monique M.A. Verstegen[3], Luc J.W. van der Laan[3], Ernst H.K. Stelzer[1], Francesco Pampaloni[1]*

[1]Physical Biology Group, Buchmann Institute for Molecular Life Sciences (BMLS), Goethe-Universität Frankfurt am Main, D-60438 Frankfurt am Main, Germany

[2]Institute of Biophysical Chemistry and Center for Biomolecular Magnetic Resonance and Cluster of Excellence Frankfurt Macromolecular Complexes (CEF-MCII), currently located at Francis Crick Institute, UK

Goethe-Universität Frankfurt am Main, D-60438 Frankfurt am Main, Germany

[3]Department of Surgery, Erasmus MC – University Medical Center, Rotterdam, The Netherlands

*corresponding author: fpampalo@bio.uni-frankfurt.de

Supplementary Material

Supplementary Figures

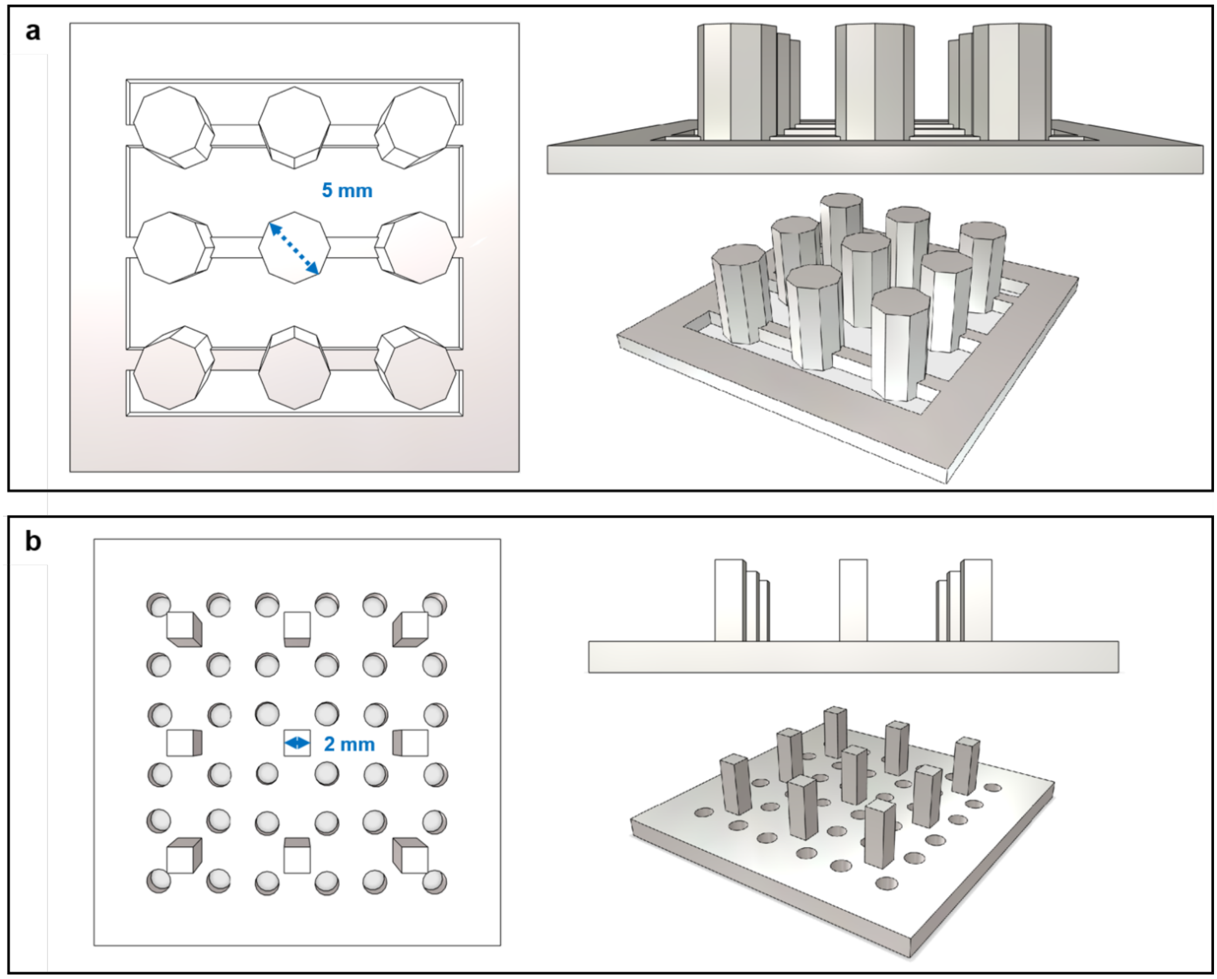

Supplementary Figure 1: CAD-derived drawings of two positive moulds for ultra-thin FEP-foil cuvettes.

Multiple cuvettes or other chambers are produced in parallel. Their number depends on their size and the extent of the base plate of the vacuum forming device. **(a)** Above, lateral and perspective views of the positive mould for ultra-thin FEP-foil cuvettes with an octagonal cross section and a diameter of 5 mm. **(b)** Comparable views of the positive mould for ultra-thin FEP-foil cuvettes with a square cross section and a width of 2 mm.

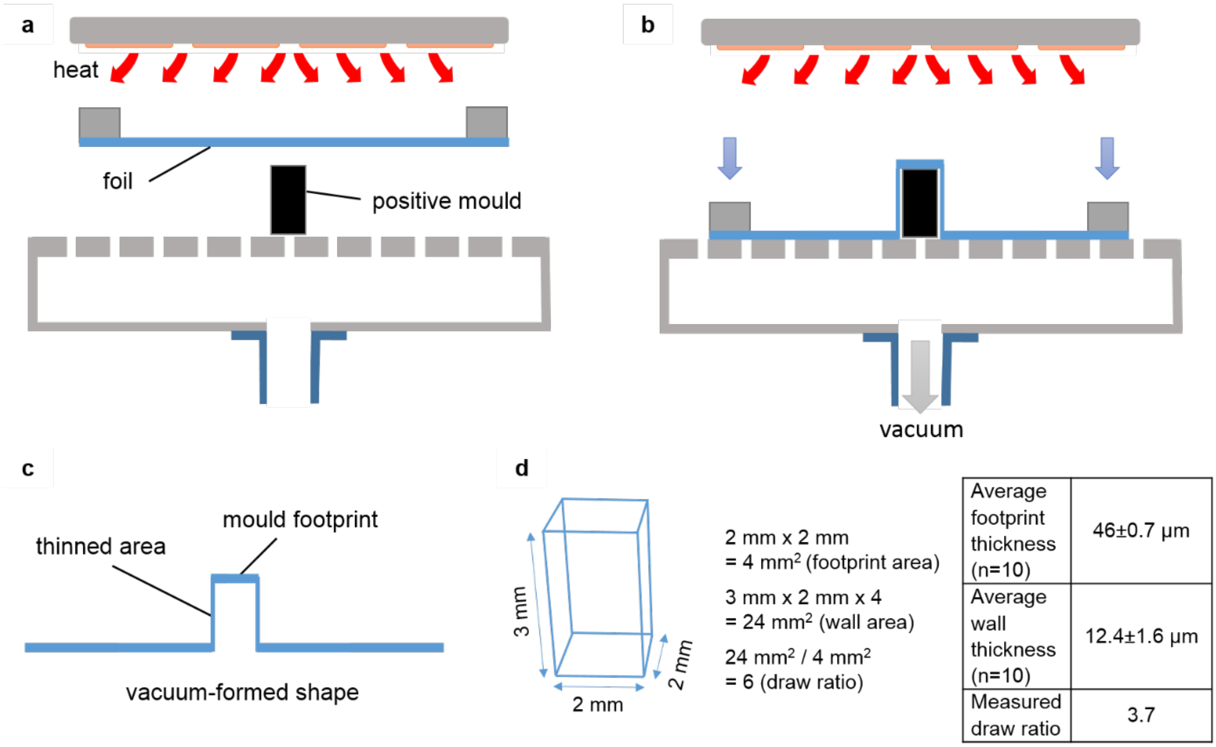

Average footprint thickness (n=10)	46±0.7 µm
Average wall thickness (n=10)	12.4±1.6 µm
Measured draw ratio	3.7

Supplementary Figure 2: Vacuum forming process for ultra-thin FEP-foil cuvettes with a square cross section.

(a) The thermoplastic FEP-foil is held firmly in a frame and heated to a target temperature close to its glass transition temperature. A positive mould is placed on the perforated vacuum plate. **(b)** Once the target temperature is achieved, the vacuum is established while the foil is stretched over the positive mould onto the perforated plate. **(c)** As soon as the foil has cooled down, the vacuum pressure is released and the ultra-thin FEP-foil cuvette is detached from the mould. **(d)** Calculation of the footprint area, the stretched wall area and the draw ratio based on the geometry of a cuvette with a square cross section. The measured average footprint thickness and stretched wall thickness shown in the table result in an experimental draw ratio of about four.

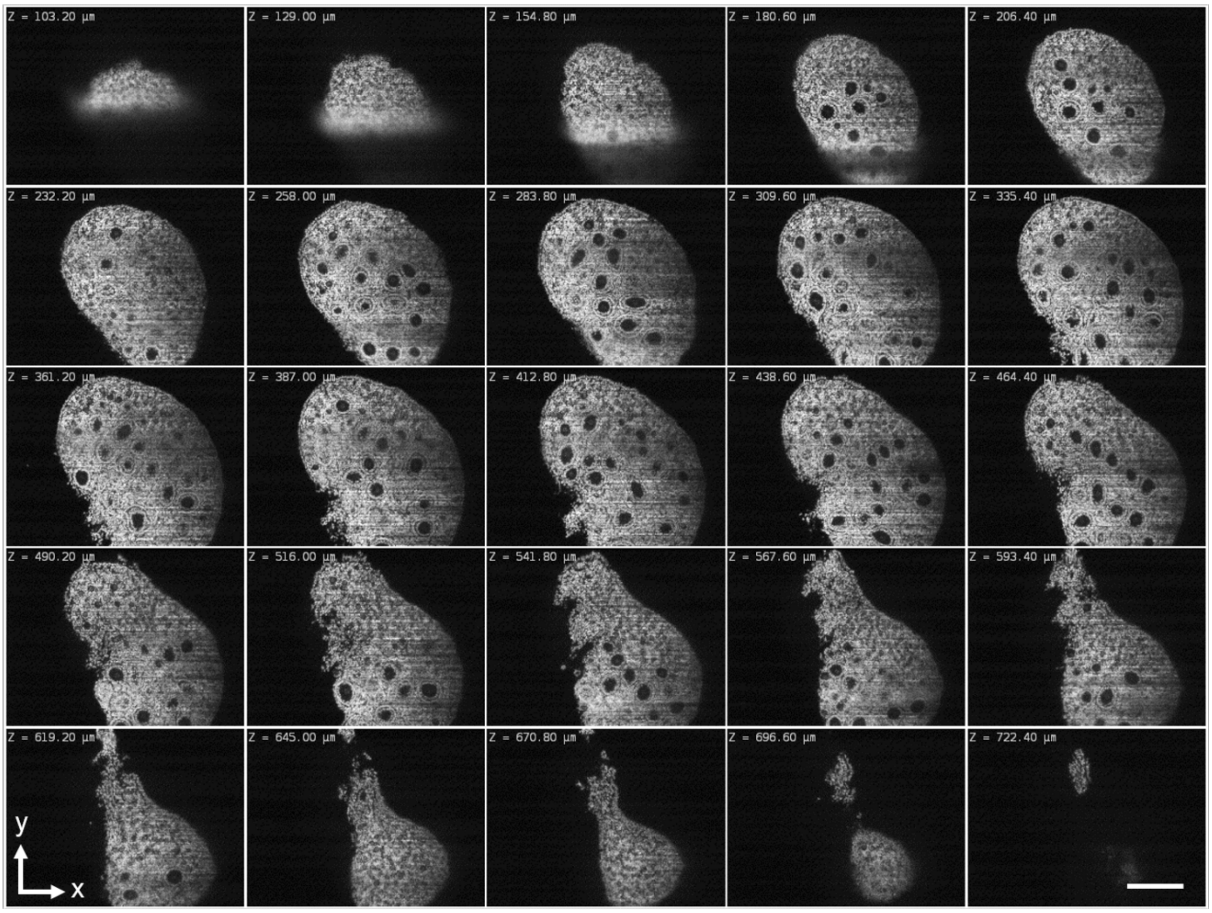

Supplementary Figure 3: Selected frames from an image stack of a murine ovary optically cleared with CUBIC2.

Clearing with CUBIC2 reduces the heterogeneity of the optical density, i.e. decreasing scattering effects and hence blur, which allows the imaging of entire ovaries. In combination with ultra-thin FEP-foil cuvettes, a penetration depth of about 700 µm is easily achieved in LSFM. Staining: cell nuclei (DAPI). Microscope: mDSLM. Objective lenses: Epiplan-Neofluar 2.5x/0.06 (excitation). N-Achroplan 10x/0.3 (detection). Excitation wavelength: 405 nm. Bandpass detection filter: 447/25 nm. Scale bar: 300 µm.

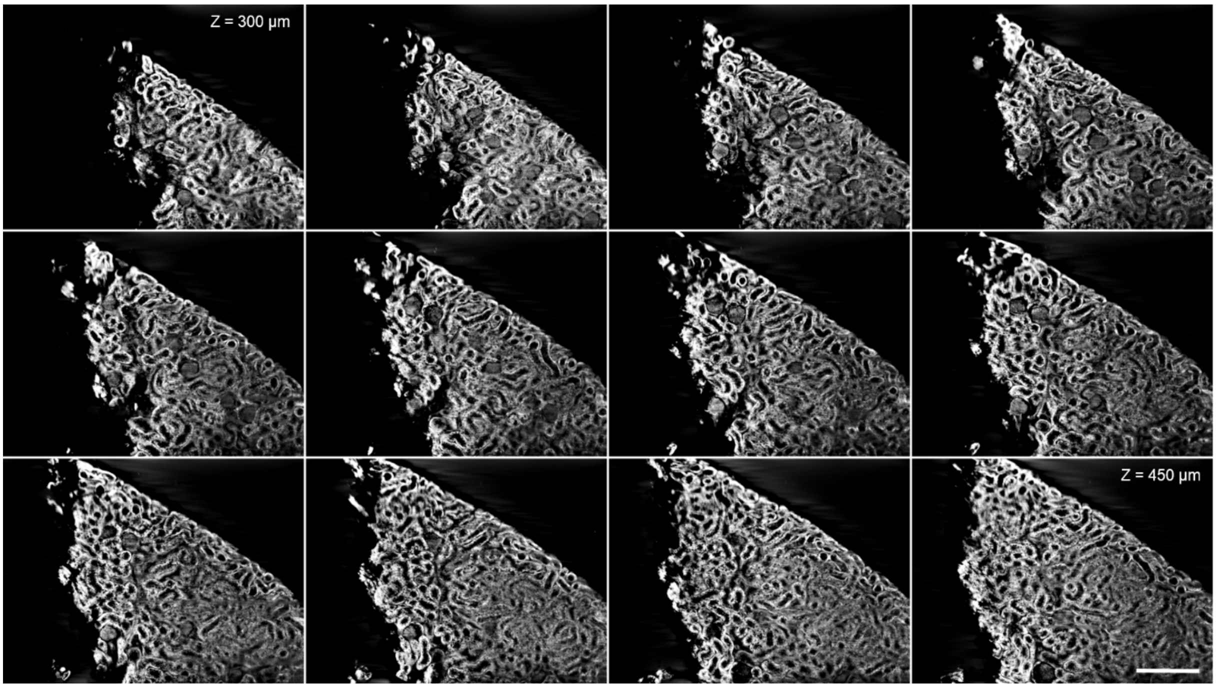

Supplementary Figure 4: Selected frames from an image stack of a murine kidney section optically cleared with ethyl cinnamate (ECi).

Images of an optically cleared murine kidney section in an ultra-thin FEP-foil cuvette. The autofluorescence signal of the kidney fragment was detected. The complex kidney ductal structure and the glomeruli are clearly visible in the images. Microscope: mDSLM. Objective lenses: Epiplan-Neofluar 2.5x/0.06 (excitation). N-Achroplan 10x/0.3 (detection). Excitation wavelength: 488 nm. Band pass detection filter: 525/50 nm. Scale bar: 200 µm.

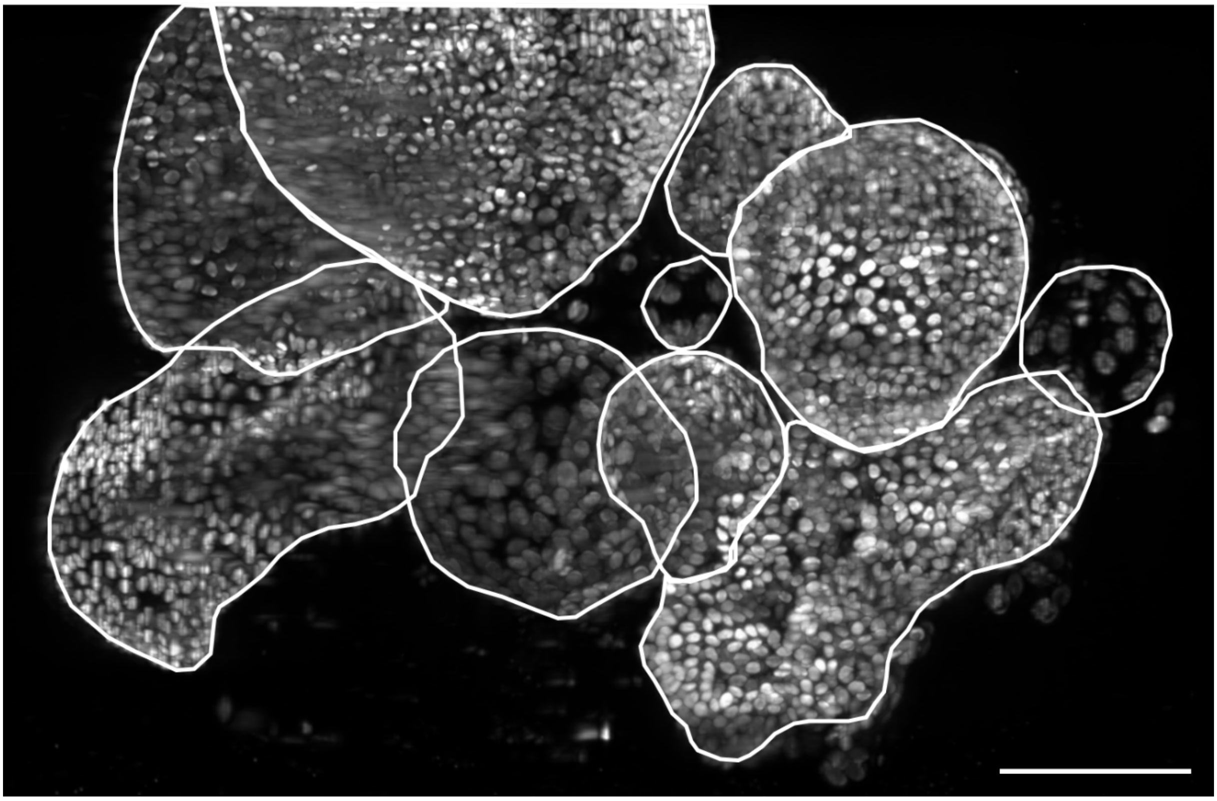

Supplementary Figure 5: Maximum intensity xy-projection of a dense cluster of human liver organoids partially embedded in Matrigel.

Maximum intensity xy-projection of a fused multiple-view dataset (0°, 90°, 180°, 270°) comprising 301 z-planes. Individual organoids are highlighted with white contour lines. Staining: cell nuclei (DAPI). Microscope: mDSLM. Objective lenses: Epiplan-Neofluar 2.5x/0.06 (excitation). N-Achroplan 10x/0.3 (detection). Excitation wavelength: 405 nm. Bandpass detection filter: 447/25 nm. Scale bar: 100 µm.

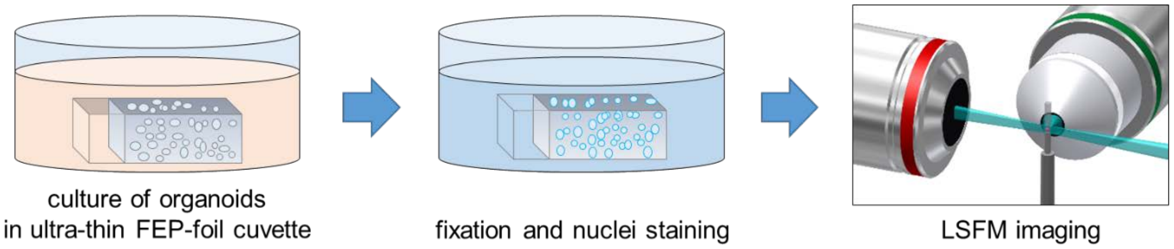

Supplementary Figure 6: Long-term culture of pancreas organoids inside ultra-thin FEP-foil cuvettes for LSFM.

Murine pancreas organoids were embedded in Matrigel and cultured inside ultra-thin FEP-foil cuvettes**.** The cuvettes were placed in a 48-well plate and covered with expansion medium to ensure growth factor and nutrient supply. The ultra-thin FEP-foil cuvettes provide a physiological-relevant gas exchange and are suitable for a culture periods of up to ten days. Since no sample transfers are required for fixation and staining, the organoid morphology remains preserved. This is a prerequisite for high quality three-dimensional imaging.

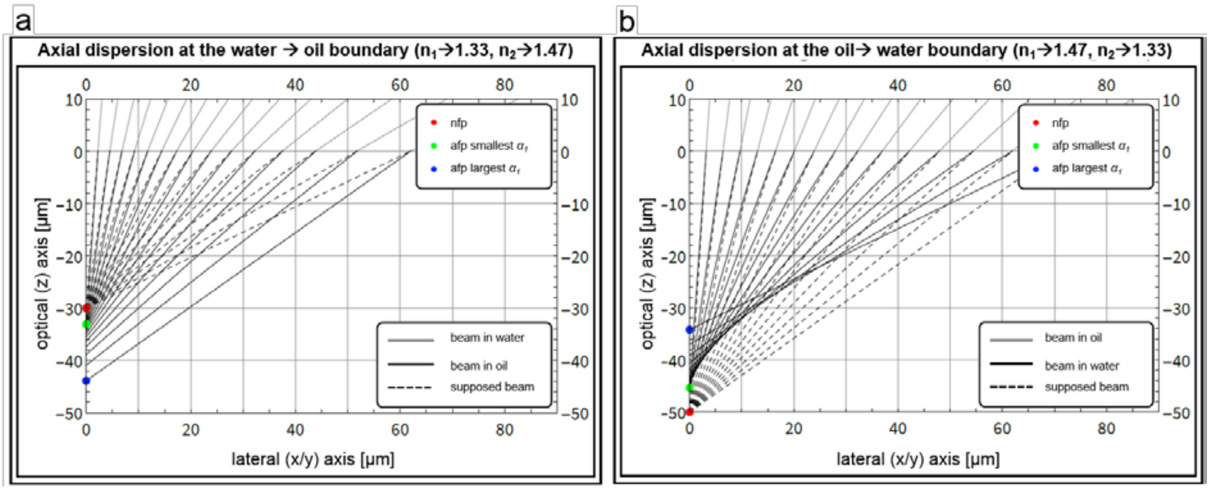

Supplementary Figure 7: Raytracing calculations show the axial dispersion and the positions of NFP and AFP along the optical axis.

Ideally, all beams focused by a microscope objective lens hit the optical axis in the same location (red dot, nfp nominal focal position). Boundaries between two media or solids with different refractive indices cause the refraction of a beam of light. Since the refraction angle is roughly proportional to the angle of the incoming beam, the intersection of the beams becomes dispersed. The green dot (afp-small actual focal position) indicates the intersection of the beams close to zero degrees, while the blue dot (afp-large) indicates the intersection of the largest angle. (a) The transition from a small to a large refractive index, e.g. from water to oil or CUBIC2, places the nfp closer to the boundary (at z=0). The dispersed beams are further inside the medium or solid with the higher refractive index. (b) The transition from a large to a small refractive index, e.g. from oil or CUBIC2 to water, places the nfp further away from the boundary (at z=0). The dispersed beams are closer to the boundary inside the medium or solid with the lower refractive index. The relation between incidence and refraction angles is given by Snell's law $n_1 \sin \alpha_1 = n_2 \sin \alpha_2$. By applying Snell's law and simple trigonometric algebra, we can verify that the AFP is directly proportional to the thickness d, the refractive index ratio n_1/n_2, and the cosine of the angle of incidence $\cos \boldsymbol{\alpha_1}$: $f \sim (d,\ n_1/n_2,\ \cos \boldsymbol{\alpha_1})$.

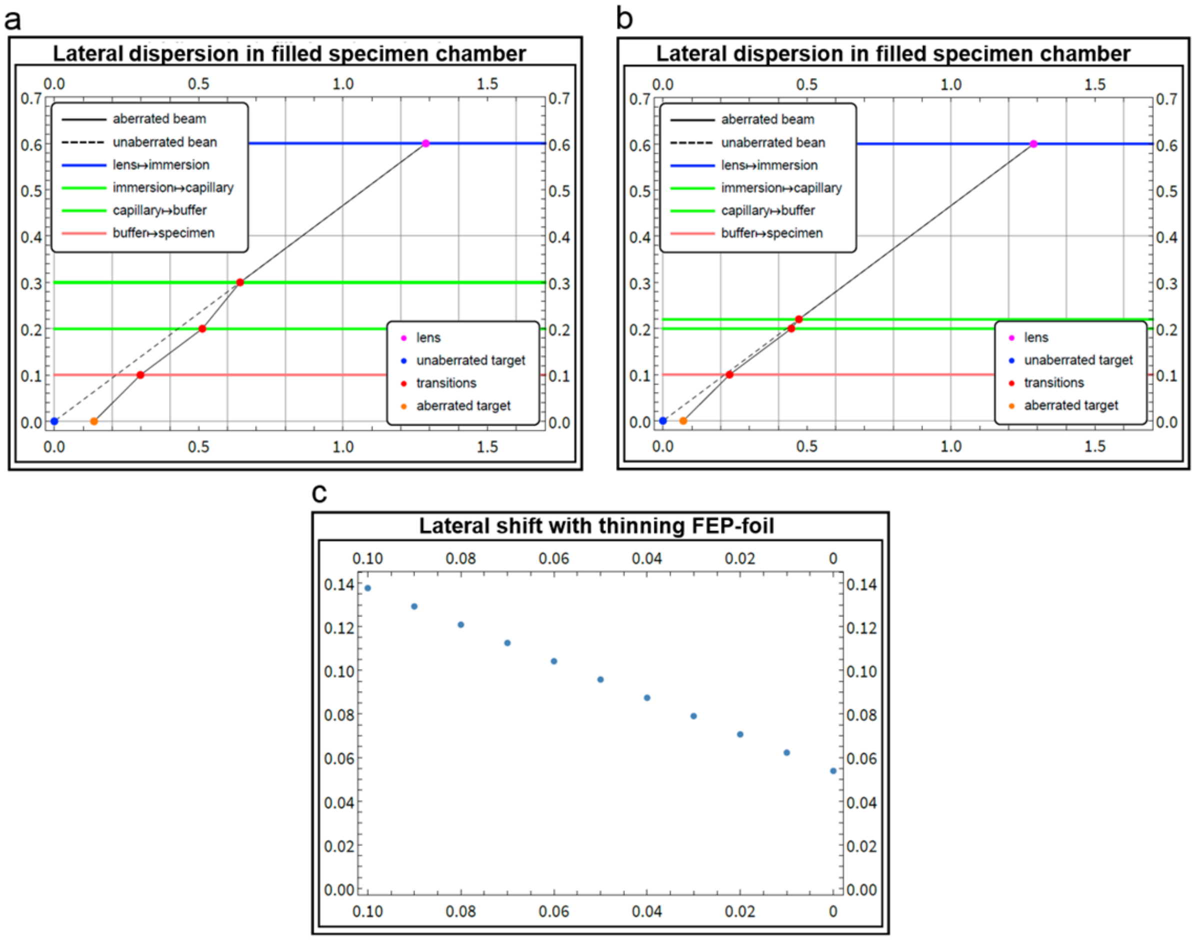

Supplementary Figure 8: Raytracing calculations show the lateral dispersion due to a chamber.

Ideally, all beams focused by a microscope objective lens hit the optical axis in the same location (blue dot). However, the heterogeneity definitely encountered with live specimens and quite often with cleared specimens, disperses the beam. The three figures show the effects of different chamber/cuvette/capillary thicknesses. (a,b) The thicker the wall (green-green spacing), the larger is the distance between the blue and the orange locations. (c) As the wall thickness becomes smaller, the shift between the locations indicated by the blue and orange dots diminishes.

Supplementary Tables

Supplementary Table 1: Raw data of measured Full Width Half Maximum (FWHM) values of fluorescent Tetraspeck beads embedded in PBS and CUBIC2, excited with 488 nm and imaged with an mDSLM. The expected values for the lateral and axial FWHMs are 1.8 µm and 14 µm respectively.

	**PBS (XY) [µm]**	**CUBIC2 (XY) [µm]**	**PBS (YZ) [µm]**	**CUBIC2 (YZ) [µm]**
**Bead 01**	2.37	1.31	14.49	8.75
**Bead 02**	2.26	1.64	13.69	8.87
**Bead 03**	2.27	1.35	13.55	11.52
**Bead 04**	2.24	1.64	15.32	10.73
**Bead 05**	2.63	1.27	16.79	8.68
**Bead 06**	1.91	1.08	13.69	10.32
**Bead 07**	1.73	1.19	14.73	11.29
**Bead 08**	1.98	1.99	13.28	8.87
**Bead 09**	2.05	2.31	13.71	8.87
**Bead 10**	1.79		12.74	8.75
**mean**	2.15	1.5	14.2	9.78
**standard deviation**	0.26	0.379	1.12	1.11
**Improvement**	(2.15±0.26 – 1.5±0.38)/2.15±0.26 = (30±22)%		(14.2±1.12 – 9.8±1.11)/14.2±1.12 = (31±11)%	

Supplementary Table 2: Summary of the morphological features drawn from the nuclei segmentation of the three different organoids displayed in Figure 5, pixel size: 0.654 x 0.654 µm², voxel size: 0.654 x 0.654 x 0.654 µm³.

	**Organoid No. 1**	**Organoid No. 2**	**Organoid No. 3**
**NucleiCount [#]**	823	803	261
**Volume [# of voxels]**	89605859	57111992	24797363
**SurfaceArea [# of pixels]**	993789	788656	423386
**MinCount [# of voxels]**	500	501	504
**MaxCount [# of voxels]**	16572	11959	14681
**MeanCount [# of voxels]**	4031	3252	3925
**StandardDeviationCount [# of voxel]**	2284	1848	2664

Supplementary Table 3: Thermoplastic polymer foils commercially available for vacuum-forming.

Material	Commercial name	Refractive index	Available foil thickness
**Cycloolefin polymer**	ZEONEX, ZEONOR	1.52	40 µm - 188 µm
**Tetrafluorethylene, perfluorpropylene, FEP**	LUMOX,<br>DuPont FEP film	1.34	12.5 µm, 25 µm, 50 µm, 125 µm
**Polyamide**	GRILAMID TR	1.54	not available as foil off-the-shelf
**Polyimide**	KAPTON	1.66	from 7.9 µm to 152 µm

Movie captions

Movie 1: Three-dimensional surface rendering of an optically cleared murine ovary. Nuclei (grey) are stained with DAPI, oocytes (green) specifically express GFP-c-Kit. 360° rotation with 1° increments.

Movie 2: Three-dimensional maximum projection of an optically cleared hippocampus of a mouse expressing Thy1-GFP (green). 360° rotation with 10° increments.

Movie 3: Three-dimensional surface rendering of a native, immuno-fluorescence labelled human pancreas organoid. DAPI-stained nuclei (grey), phalloidin-stained F-actin (green), immuno-stained Sox9 (magenta).

Movie 4: Three-dimensional projection of a fused multiple-view dataset of human liver organoids in Matrigel. DAPI-stained nuclei (grey), 360° rotation with 10° increments.

Movie 5: Three-dimensional surface rendering of segmented nuclei in a murine pancreas organoid. Colours represent individual detected nuclei.

Supplementary Video

Supplementary Video 1: Image stack of a murine kidney optically cleared with ethyl cinnamate (ECi). Images of an optically cleared murine kidney section in an ultra-thin FEP-foil cuvette. The autofluorescence signal of the kidney fragment was detected. The complex kidney ductal structure and the glomeruli are clearly visible in the images. Microscope: mDSLM. Objective lenses: Epiplan-Neofluar 2.5x/0.06 (excitation). N-Achroplan 10x/0.3 (detection). Excitation wavelength: 488 nm. Band pass detection filter: 525/50 nm. Scale bar: 200 µm. Step size along the Z-axis: 2.6 µm.
